# New Insights for RANKL as a Proinflammatory Modulator in Modeled Inflammatory Arthritis

**DOI:** 10.3389/fimmu.2019.00097

**Published:** 2019-02-05

**Authors:** Maria Papadaki, Vagelis Rinotas, Foteini Violitzi, Trias Thireou, George Panayotou, Martina Samiotaki, Eleni Douni

**Affiliations:** ^1^Laboratory of Genetics, Department of Biotechnology, Agricultural University of Athens, Athens, Greece; ^2^Division of Immunology, Biomedical Sciences Research Center “Alexander Fleming”, Athens, Greece; ^3^Division of Molecular Oncology, Biomedical Sciences Research Center “Alexander Fleming”, Athens, Greece

**Keywords:** RANKL, TNF, inflammation, arthritis, transgenic models, proteomics

## Abstract

Receptor activator of nuclear factor-κB ligand (RANKL), a member of the Tumor Necrosis Factor (TNF) superfamily, constitutes the master regulator of osteoclast formation and bone resorption, whereas its involvement in inflammatory diseases remains unclear. Here, we used the human TNF transgenic mouse model of erosive inflammatory arthritis to determine if the progression of inflammation is affected by either genetic inactivation or overexpression of RANKL in transgenic mouse models. TNF-mediated inflammatory arthritis was significantly attenuated in the absence of functional RANKL. Notably, TNF overexpression could not compensate for RANKL-mediated osteopetrosis, but promoted osteoclastogenesis between the pannus and bone interface, suggesting RANKL-independent mechanisms of osteoclastogenesis in inflamed joints. On the other hand, simultaneous overexpression of RANKL and TNF in double transgenic mice accelerated disease onset and led to severe arthritis characterized by significantly elevated clinical and histological scores as shown by aggressive pannus formation, extended bone resorption, and massive accumulation of inflammatory cells, mainly of myeloid origin. RANKL and TNF cooperated not only in local bone loss identified in the inflamed calcaneous bone, but also systemically in distal femurs as shown by microCT analysis. Proteomic analysis in inflamed ankles from double transgenic mice overexpressing human TNF and RANKL showed an abundance of proteins involved in osteoclastogenesis, pro-inflammatory processes, gene expression regulation, and cell proliferation, while proteins participating in basic metabolic processes were downregulated compared to TNF and RANKL single transgenic mice. Collectively, these results suggest that RANKL modulates modeled inflammatory arthritis not only as a mediator of osteoclastogenesis and bone resorption but also as a disease modifier affecting inflammation and immune activation.

## Introduction

Receptor Activator of Nuclear Factor κB Ligand (RANKL), a Tumor necrosis factor (TNF) superfamily member, is the master regulator of osteoclast-induced bone resorption (1), that is necessary for the lifelong process of bone remodeling where mature bone tissue is removed from the skeleton and new bone tissue is formed by osteoblasts. RANKL binds as a trimer to its receptor RANK to promote osteoclast differentiation, activity and survival, which subsequently leads to bone resorption (2, 3). Osteoclasts derive from the myeloid lineage and have the unique ability to resorb bone through the decalcification and degradation of the bone matrix by hydrochloric acid and proteolysis, respectively (4). Genetic ablation of either RANKL or RANK results in severe osteopetrosis, a disease caused by osteoclast deficit, demonstrating that the RANKL/RANK system is indispensable for osteoclastogenesis (5–7). The function of RANKL is physiologically inhibited by the action of the decoy receptor osteoprotegerin (OPG) that binds to RANKL and prevents the process of osteoclastogenesis (8). An imbalance at the RANKL:OPG ratio caused by abundant RANKL levels is believed to be a major determinant in the development of bone loss diseases, including postmenopausal osteoporosis, a metabolic bone disease characterized by decreased bone density and increased fracture risk (9). The critical role of RANKL in osteoporosis is now well-established by the efficacy of denosumab, a human monoclonal anti-RANKL antibody, that specifically inhibits the interaction between RANKL and RANK, in postmenopausal osteoporosis (10). Although RANKL is best known for its function in osteoclastogenesis, it also plays multiple roles in the immune system (11), as it has been shown to enhance dendritic cell survival and regulates lymph node organogenesis. In addition, RANKL controls the development of autoimmune regulator (AIRE)^+^ medullary thymic epithelial cells suggesting a key role of RANKL/RANK signals in the regulation of central tolerance. RANKL expression could also be detected in synovial fibroblasts and inflammatory cells isolated from the synovial fluid of Rheumatoid Arthritis (RA) patients, facilitating osteoclast maturation even in the absence of osteoblasts. Although the inhibition of RANKL effectively arrests progression of arthritic osteolysis, there are no evidence so far to support proinflammatory properties of RANKL (12). Thus, the role of RANKL in the progression of inflammation in RA remains unclear.

RA is a complex inflammatory disease characterized by synovial hyperplasia, cartilage damage, and bone erosions, leading to progressive disability. Inflammatory synovium, mainly including macrophage-like and fibroblast-like synoviocytes, leads to pannus formation that destroys the local articular structures through proteolytic digestion of the extracellular matrix (13). The destructive processes in RA involve a complex interplay between synovial fibroblasts, lymphocytes, macrophages, proinflammatory cytokines, and chemokines, inducing osteoclast-mediated bone resorption. TNF is a key proinflammatory cytokine in RA (14), as experimentally shown by the spontaneous development of chronic inflammatory polyarthritis upon TNF overexpression in transgenic mice (15, 16) and clinically by the efficacy of anti-TNF therapies in RA patients (17). Apart from its proinflammatory role, TNF also promotes bone resorption at sites of chronic inflammation, through the induction of osteoclastogenesis (18). Even though the RANK/RANKL signaling is also involved in local osteolysis induced by chronic inflammation, it remains unclear whether it is the absolute pathway. Previous studies have shown that proinflammatory cytokines such as TNF can compensate for RANKL during osteoclastogenesis *in vitro* (19–21), whereas it is unclear whether TNF can lead to osteoclastogenesis independently of RANKL, *in vivo*.

In the present study, we investigated the role of RANKL as a disease modifier in TNF-driven inflammatory arthritis employing two proprietary genetic models of RANKL-mediated pathologies; an osteopetrosis model caused by osteoclast absence due to a functional mutation in the *RANKL* gene (22) (Rankl^tles/tles^ mice) and osteoporosis transgenic models that overexpress human RANKL (TgRANKL mice) displaying increased osteoclast activity and bone resorption (23). Our results showed that the onset and the progression of TNF-mediated arthritis is dramatically affected by deregulated RANKL expression, supporting an underestimated role of RANKL in inflammatory osteolytic diseases.

## Materials and Methods

### Mouse Husbandry

Osteopetrotic Rankl^tles/tles^ mice (22), osteoporotic Tg5516 and Tg5519 mice (23), and arthritic Tg197 mice (15) were maintained and bred under specific pathogen free conditions in the animal facility of Biomedical Sciences Research Center “Alexander Fleming.” All animal procedures were approved and carried out in strict accordance with the guidelines of the Institutional Animal Care and Use Committee and the Region of Attica Veterinarian Office.

### Arthritic Clinical Score

Arthritis was evaluated macroscopically weekly in ankle joints in a blinded manner using the following semi-quantitative arthritis score (24); 0: no arthritis (normal appearance and grip strength); (1) mild arthritis (joint swelling); (2) moderate arthritis (severe joint swelling and digit deformation, no grip strength); and (3) severe arthritis (ankylosis detected on flexion and severely impaired movement). Grip strength was evaluated as regards the ability of the mouse to grasp the cage grid cover.

### Histological Processing and Scoring of Joints

Ankle joints and femurs were fixed in 10% formalin overnight at 4°C, decalcified in 13% EDTA for 14 days, and embedded in paraffin. Sections of 5 μm thickness were stained with hematoxylin and eosin, and the histopathologic score was evaluated microscopically, in a blinded manner using a modified scoring system (24) as follows; 0: no detectable pathology; 1: hyperplasia of the synovial membrane and presence of polymorphonuclear infiltrates; 2: pannus and fibrous tissue formation and focal subchondral bone erosion; 3: articular cartilage destruction and bone erosion; 4: extensive articular cartilage destruction and bone erosion, and 5: massive destruction of ankle joint with undefined structure. Osteoclasts were stained for TRAP (Tartrate Resistant Acid Phosphatase) activity using the leukocyte acid phosphatase kit 386A (Sigma-Aldrich), whereas cartilage was stained with Toluidine Blue (Sigma-Aldrich). TRAP staining was quantified as an osteoclast surface fraction (percentage of osteoclast surface in total bone surface, Oc.S/BS, %) focusing either in the bone marrow compartment area or the pannus-bone interface area using the open source software for bone histomorphometry “TrapHisto” (25).

### MicroCT Analysis

Bone samples (ankles and femurs) were fixed in 10% formalin overnight at 4°C and then washed and stored in PBS. Microarchitecture of the ankle joints and the distal femurs from 6 weeks old mice was evaluated using a high-resolution SkyScan1172 microtomographic (microCT) imaging system (Bruker). Images were acquired at 50 KeV, 100 μA with a 0.5 mm aluminum filter. Three-dimensional reconstructions (8.8 mm cubic resolution) were generated using NRecon software (Bruker) as previously described (26). For the trabecular area of the calcaneous bone, we assessed the bone volume fraction (BV/TV, %), and trabecular number (Tb.N, mm^−1^). Calcaneous trabecular geometry was assessed using 75 continuous CT slides (300 μm) located at trabecular area underneath the growth plate of the calcaneous bone. For the trabecular area of the distal femur bone we assessed the bone volume fraction (BV/TV, %), and the trabecular number (Tb.N, mm^−1^). Femoral trabecular geometry was assessed using 300 continuous CT slides (1,800 μm) located at the trabecular area underneath the growth plate. Femural cortical geometry was assessed using 100 continuous CT slides (600 μm) located at the femoral midshaft, where the bone volume fraction (BV/TV, %) and the bone volume (Ct.BV, mm^3^) were measured.

### Flow Cytometry

Mice were sacrificed, ankle joints were removed and cells were extracted from the synovium based on a modified protocol (27). In brief, synovial tissue from ankle joints was minced in RPMI medium containing 5% FBS, glutamine and freshly made Collagenase type II isolated from *Clostridium histolyticum* (Worthington) and incubated in a shaking waterbath for 90 min at 37°C. Single cell suspensions were generated through pushing the tissue on a size-40 metallic mesh disc (Sigma-Aldrich). Cells were filtered through a 100-μm sheet, centrifuged, resuspended in FACS buffer (1% FBS in PBS) and counted using a hematocytometer. 10^6^ cells were plated in a 96 V-bottom well plate (Costar) and stained with antibodies against CD45-Alexa 700, CD11b-PE, Gr1-FITC, TCRα-APC/Cy7, and B220-PerCP (Biolegend). Cells were incubated for 30 min at 4°C, and then were washed and transferred to tubes for analysis. BD FACS Canto II Flow Cytometer (BD Biosciences) was used for processing the samples and results were analyzed with FlowJo v7.6 software.

### Quantitative Expression Analysis

Total RNA was extracted from ankle joints using a monophasic solution of guanidine isothiocyanate and phenol according to the manufacturer's instructions (TRI Reagent, MRC). After removal of DNA remnants with DNase I treatment (Sigma-Aldrich), first strand cDNA was synthesized using 2 μg of total RNA and MMLV reverse transcriptase (Sigma-Aldrich). Templates were amplified with SsoFast EvaGreen Master Mix (Bio-Rad Laboratories) on the CFX96 real time PCR instrument (Bio-Rad Laboratories). Quantitative Real Time PCR (qPCR) was performed at 55°C for all genes (except: IL6 at 58°C) for 40 cycles. Specific primer pairs (Eurofins Genomics) were used for the quantitative expression as follows (sequences 5′ to 3′, sense and antisense): *human RANKL:* ACGCGTATTTACAGCCAGTG and CCCGTAATTGCTCCAATCTG; *mouse RANKL:* TGTACTTTCGAGCGCAGATG and AGGCTTGTTTCATCCTCCTG; *human TNF:* GAGGCCAAGCCCTGGTATG and CGGGCCGATTGATCTCAGC; *mouse TNF:* CAGGCGGTGCCTATGTCTC and CGATCACCCCGAAGTTCAGTAG; *mouse IL-1*β*:* ATCTTTTGGGGTCCGTCAACT and CCCTCACACTCAGATCATCTTCT; and *mouse IL-6*: TAGTCCTTCCTACCCCAATTTCC and TTGGTCCTTAGCCACTCCTTC. The samples were normalized to GAPDH expression (TTAGCACCCCTGGCCAAGG and CTTACTCCTTGGAGGCCATG). Relative expression was calculated as the fold difference compared with control values using BioRad CFX96^TM^. For each experiment at least three biological and two technical replicates were used.

### Proteomics

For the proteomic analysis, ankle joints were isolated from 6-week-old WT, Tg5519, Tg197 and Tg197/Tg5519 mice (6–8 mice per genotype).

#### Protein Extraction and Lysis

Ankle joints from the four different genotypes (WT, Tg5519, Tg197, and Tg197/Tg5519) were ground to powder in liquid nitrogen using a pestle and mortar and solubilized in 150 μl lysis buffer containing 100 mM Tris-HCl, pH 7.6, 4% SDS and freshly made 100 mM DTT. Samples were incubated for 3 min at 95°C, followed by 20 min incubation in a sonication water bath in order to shear the DNA. Finally, the samples were centrifuged at 17,000 × g for 30 min at 4°C and the supernatants were transferred to new tubes.

#### Protein Digestion

The protein extracts were processed according to the Filter Aided Sample Preparation (FASP) protocol using spin filter devices with 10 kDa cutoff (Sartorius, VN01H02). 40 μl lysate were diluted in 8 M Urea/100 mM Tris-HCl pH 8.5, the filters were extensively washed with the urea solution, covered with 10 mg/ml iodoacetamide in the urea solution and incubated for 30 min in the dark for the alkylation of cysteines. The proteins on the top of the filters were washed three times with 50 mM ammonium bicarbonate and finally the proteins were digested adding 1 μg trypsin/LysC mix in 80 μl 50 mM ammonium bicarbonate solution (Mass spec grade, Promega) and incubated overnight at 37°C. The peptides were eluted by centrifugation, followed by speed-vac-assisted solvent removal, reconstitution in 0.1% formic acid, 2% acetonitrile in water, and transferring to LC-MS glass sample vials. Peptide concentration was determined by nanodrop absorbance measurement at 280 nm.

#### Ultra High Pressure NanoLC

2.5 μg peptides were injected and pre-concentrated with a flow of 3 μl/ min for 10 min using a C18 trap column (Acclaim PepMap100, 100 μm × 2 cm, Thermo Scientific) and then loaded onto a 50 cm C18 column (75 μm ID, particle size 2 μm, 100 Å, Acclaim PepMap RSLC, Thermo Scientific). The binary pumps of the HPLC (RSLCnano, Thermo Scientific) consisted of solution A (2% v/v ACN in 0.1% v/v formic acid) and solution B (80% ACN in 0.1% formic acid). The peptides were separated using a linear gradient of 4–40% B in 450 min at a flow rate of 300 nl/min. The column was placed in an oven operating at 35°C.

#### MS/MS

The purified peptides were ionized through nanoESI and analyzed by an LTQ Orbitrap XL Mass spectrometer (Thermo Fisher Scientific). Full scan MS spectra were acquired in the orbitrap (*m*/*z* 300–1,600) using profile mode with a data-dependent acquisition method were the resolution was set to 60,000 at *m*/*z* 400 and the automatic gain control target at 10^6^ ions. The six most intense ions were sequentially isolated for collision-induced MS/MS fragmentation and their detection in the linear ion trap. Dynamic exclusion was set to 1 min and activated for 90 sec. Ions with single charge states were excluded. Lockmass of m/z 445.120025 was used for internal calibration. Xcalibur (Thermo Scientific) was used to control the system and acquire the raw files.

#### Data Analysis

The raw files were analyzed using MaxQuant (version 1.6.0.16), the complete Uniprot *Mus musculus (228 311 entries / Oct-2016)* and a common contaminants database by the Andromeda search engine. The search parameters used were strict trypsin specificity, allowing up to two missed cleavages. Oxidation of methionines, deamidation of asparagine and glutamine residues and N-terminal acetylation were set as variable modifications. Cysteine carbamidomethylation was set as a fixed modification. “Second peptide” option was enabled. The protein and peptide false discovery rate (FDR) was set to 0.01 for both proteins and peptides with a minimum length of seven amino acids that was determined by searching a reverse database. Protein abundance was calculated on the basis of the normalized spectral protein intensity as label free quantitation (LFQ intensity) enabling the “match between runs” option (set at 0.7 min). LFQ was performed with a minimum ratio count of 2.

#### Proteomics Statistical Analysis

The statistical analysis was performed using Perseus (version 1.6.1.3) (28). Proteins identified as contaminants, “reverse” and “only identified by site” were filtered out. The LFQ intensities were transformed to logarithmic values (log2(x)). The biological replicas were grouped together. The protein groups were filtered to obtain at least 4 valid values in at least one group. A total of 2,009 label free quantified proteins were subjected to statistical analysis with ANOVA test (permutation based FDR with 0.05 cutoff) for the comparison of all groups. The 1,019 statistically significant proteins were then Z-scored, visualized by Euclidean hierarchical clustering and grouped into three main clusters (I, II and III) consisting of 403, 179, and 437 proteins, respectively. Tukey's honestly significant difference (THSD) was performed on the ANOVA significant hits to determine in exactly which pairwise group comparisons, a given protein was differentially expressed. Enrichment analysis was performed with ClueGO (29) (version 3.6.1), a Cytoscape plug-in, using KEGG (30) pathways database. Only pathways that had *p*-value < 0.05 (hypergeometric test with Benjamini–Hochberg correction) were considered and “is Specific” was set to 60%. Default values were used for the other parameters.

### Statistical Analysis

All results are expressed as mean ± standard error mean (SEM). Statistical significance was calculated for two groups using Student's *t*-tests or the Mann-Whitney test for non-parametric distribution. The log-rank test was used for survival curve comparison. One-Way analysis of variance (ANOVA) and Tukey *post-hoc* test was performed to compare means of multiple groups. *P*-values < 0.05 were considered significant; ^*^p < 0.05, ^**^p < 0.01, ^***^p < 0.001 when not otherwise specified.

## Results

### Significant Attenuation of TNF-Mediated Inflammatory Arthritis in the Absence of Functional RANKL

**Figure 1:**
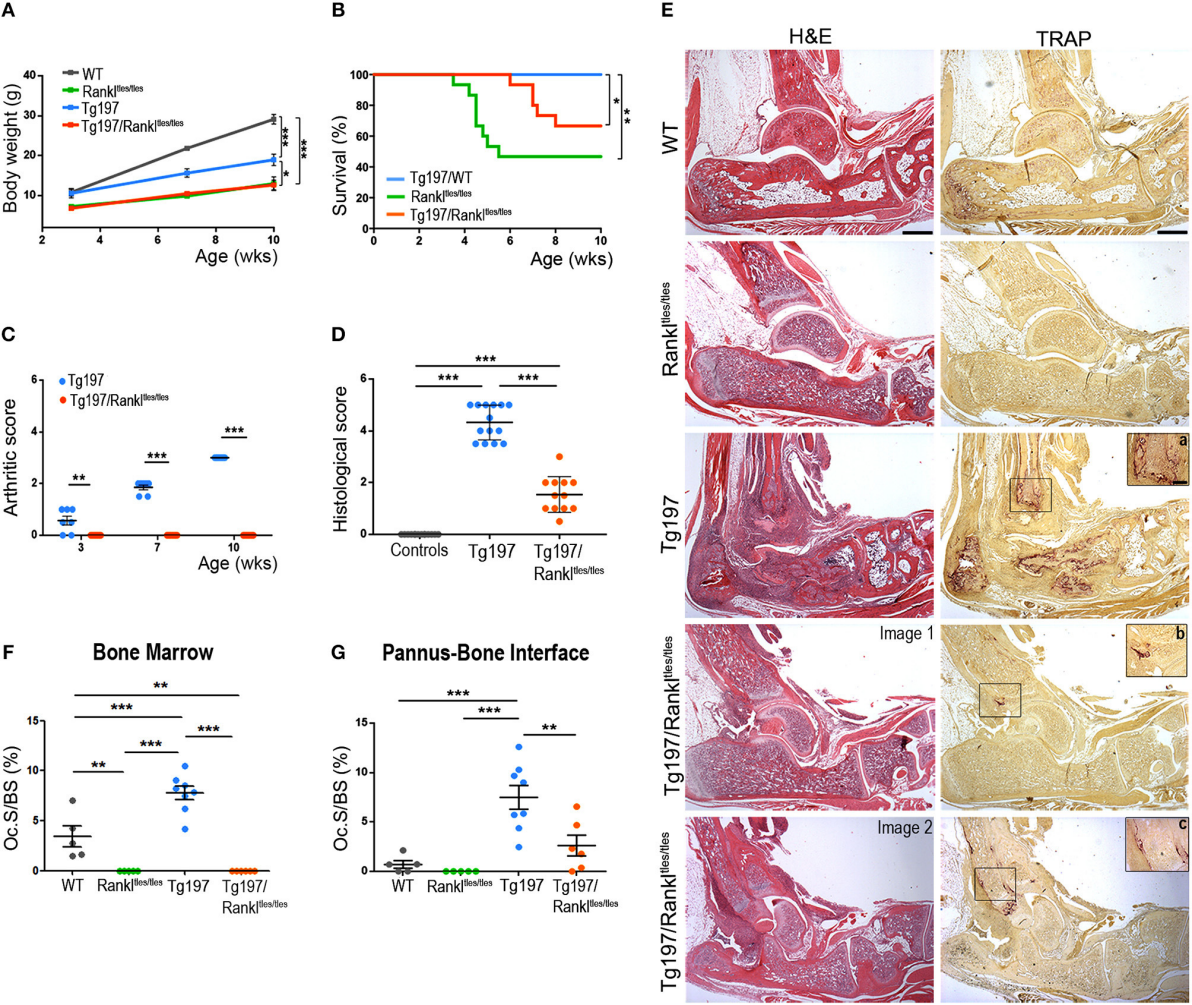
Dramatic attenuation of TNF-driven arthritis upon RANKL genetic inactivation. Tg197/Rankl^tles/tles^ mice and sex-matched control littermates WT (Rankl^tles/+^), Tg197 (Tg197/Rankl^tles/+^), and Rankl^tles/tles^ were assessed until the 10th week of age for **(A)** body weight gain (*n* = 6–7 per genotype), **(B)** percent survival (*n* = 15 per genotype), **(C)** clinical arthritic score (from 0 to 3) in both ankles for each mouse (*n* = 7 per genotype), and **(D)** histological arthritic score (from 0 to 5) in both ankles for each mouse at 10 weeks of age (*n* = 12–14 per genotype). Control group includes Rankl^tles/+^ and Rankl^tles/tles^ mice. **(E)** Representative histological images of hematoxylin/eosin (H&E) and Tartrate-resistant acid phosphatase (TRAP) stained ankle joint sections from two Tg197/Rankl^tles/tles^ mice, displaying either mild (Image 1), or moderate inflammatory arthritis (Image 2), and their littermate controls at 10 weeks of age. Boxed areas at TRAP staining show a higher magnification of regions harboring TRAP+ cells in Tg197 **(a)** and Tg197/Rankl^tles/tles^ mice **(b,c)**. Scale bars: 300 μm at H&E and TRAP, 80 μm at boxed areas in TRAP. TRAP staining was measured as osteoclast surface fraction (Oc.S/BS, %) quantification in **(F)** the bone marrow compartment area, and **(G)** the pannus-bone interface (*n* = 5–8 per genotype). Data represent mean values ± SEM. One-Way ANOVA and Tukey *post-hoc* test was performed for statistical analysis of more than two groups and Mann-Whitney test was performed for statistical analysis between two groups. The log-rank test was used for survival curve comparison. Asterisks mark statistically significant difference (^*^p < 0.05, ^**^p < 0.01, ^***^p < 0.001).

To elucidate the role of RANKL in the progression of TNF-mediated inflammatory arthritis *in vivo*, we generated Tg197/Rankl^tles/tles^ mice by crossing Tg197 arthritic mice overexpressing human TNF with Rankl^tles/tles^ osteopetrotic mice carrying a functional mutation in the *RANKL* gene (22). The Tg197 transgenic mouse model spontaneously develops inflammatory arthritis characterized by swelling of the ankles, infiltration of inflammatory cells, synovial hyperplasia, articular cartilage destruction and bone erosion, closely resembling the human pathology of rheumatoid arthritis. Rankl^tles/tles^ mice, expressing an inactive form of RANKL incapable of forming trimers, are osteopetrotic due to osteoclast absence (22). Tg197/Rankl^tles/tles^ mice also displayed an osteopetrotic phenotype as shown by failure of tooth eruption, and growth retardation similarly to Rankl^tles/tles^ mice, whereas an improvement was observed in their survival percentage compared to Rankl^tles/tles^ mice even though not significant (Figures 1A,B). Macroscopically, arthritis appeared in Tg197 mice at 3 weeks of age as detected by mild swelling of the ankle joint, which progressed with severe joint swelling and distortion accompanied by movement deterioration by the 10th week of age, the end point of the study (Figure 1C). However, arthritis signs were not detected in Tg197/Rankl^tles/tles^ mice throughout the study period (Figure 1C). Histological analysis at 10 weeks of age, when Tg197 control mice reached the peak of disease, demonstrated a dramatic attenuation of inflammatory arthritis in Tg197/Rankl^tles/tles^ mice, as shown by moderate synovial hyperplasia (Figures 1D,E). These results indicate that RANKL loss significantly attenuates inflammatory arthritis onset and progression.

### RANKL-Independent Formation of Osteoclasts in TNF-Driven Inflammatory Arthritis

So far, it has been shown that RANKL is necessary for the physiological process of bone remodeling. However, it is unclear whether osteoclasts can be formed in a TNF-driven inflammatory environment even without RANKL signaling *in vivo*. To investigate this possibility, we analyzed the hematoxylin/eosin stained sections for osteopetrosis and in parallel we stained serial sections from all experimental groups with Tartrate-resistant acid phosphatase (TRAP), which is an osteoclastic marker. As expected, Rankl^tles/tles^ mice failed to develop TRAP+ osteoclasts and developed osteopetrosis, whereas enhanced osteoclastogenesis and bone resorption was identified in arthritic Tg197 mice at sites of pannus invasion into bone (Figures 1E–G). However, TNF overexpression failed to reverse the RANKL-mediated osteopetrotic phenotype in Tg197/Rankl^tles/tles^ mice, which was further confirmed by the absence of osteoclastogenesis in the bone marrow compartment (Figures 1E,F). Instead, TRAP+ osteoclasts were identified in the inflamed synovium of Tg197/Rankl^tles/tles^ mice (Figures 1E,G), indicating RANKL-independent mechanisms of osteoclastogenesis at sites of TNF-induced inflammation *in vivo*. Notably, the extent of osteoclastogenesis, either limited or moderate, depended on arthritis severity in Tg197/Rankl^tles/tles^ ankles (Figures 1E,G). Collectively, our results suggest that TNF overexpression can induce RANKL-independent osteoclastogenesis at sites of inflammatory invasion into the ankle joints but cannot compensate for RANKL in bone remodeling *in vivo* as the osteopetrotic phenotype is not affected.

### RANKL Overexpression Exacerbates TNF-Driven Inflammatory Arthritis

**Figure 2:**
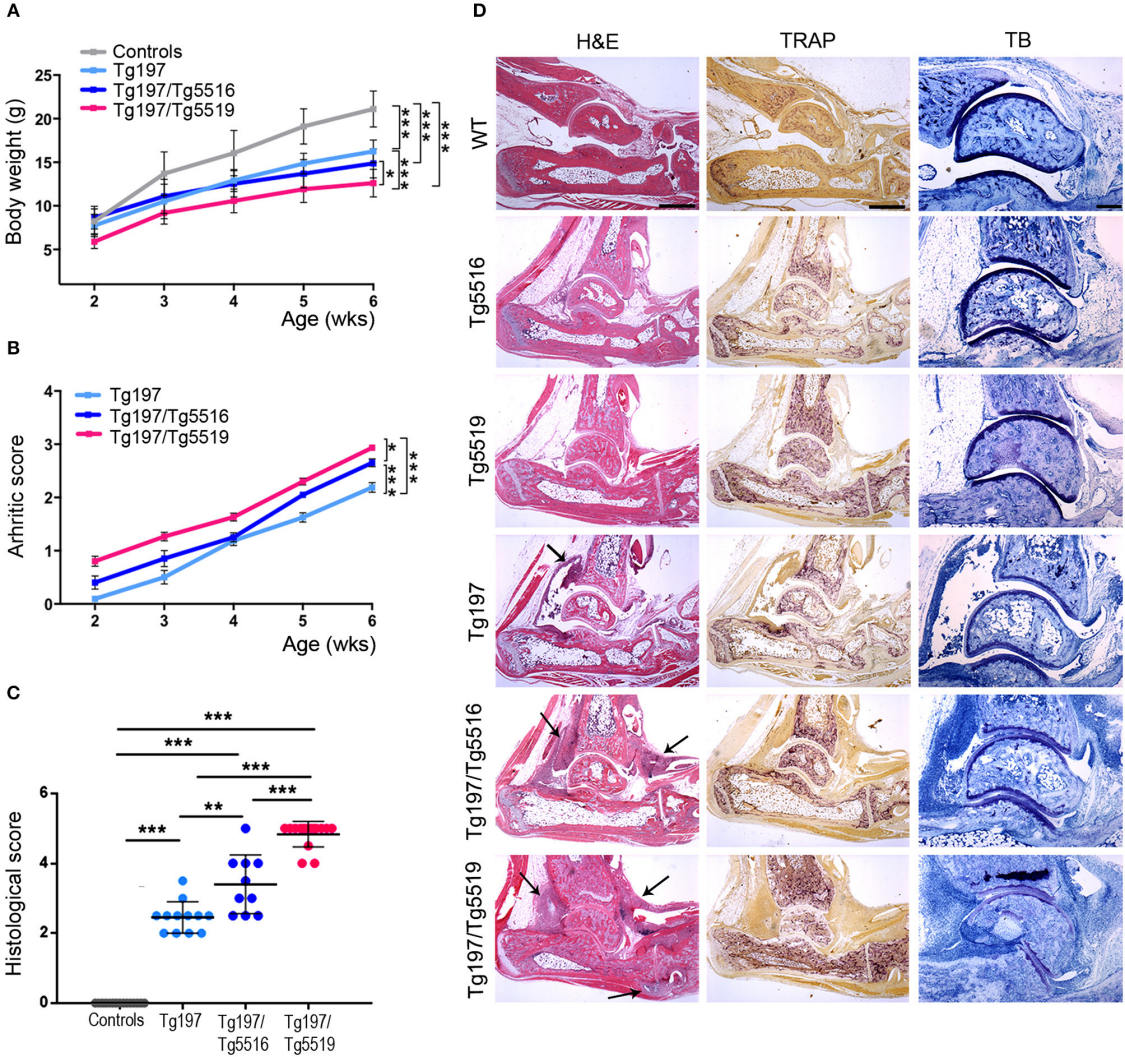
RANKL overexpression exacerbates TNF-driven arthritis. Assessment of arthritis progression was conducted in Tg197/TgRANKL mice (Tg197/Tg5516, Tg197/Tg5519), Tg197 and sex-matched control littermates (WT, Tg5516, and Tg5519) till the 6th week of age. **(A)** Body weight curves (*n* = 10 per genotype), **(B)** clinical arthritic scores (*n* = 10–15 per genotype), **(C)** histological arthritic score (*n* = 10–15 per genotype), and **(D)** representative ankle joint sections from each genotype (*n* = 10–15) at the 6th week of age stained with hematoxylin/eosin (H&E), TRAP for osteoclasts and Toluidine Blue (TB) for articular cartilage destruction. Arrows at H&E indicate focal pannus invasion into subchondral bone regions. Scale bars: 300 μm at H&E and TRAP; 150 μm at TB. Data represent mean values ± SEM. One-Way ANOVA and Tukey *post-hoc* test was performed. Asterisks mark statistically significant difference (^*^p < 0.05, ^**^p < 0.01, ^***^p < 0.001).

We next investigated whether the progression of inflammatory arthritis in the TNF transgenic model was affected by human RANKL (huRANKL) overexpression. This was achieved by crossing Tg197 mice with the TgRANKL transgenic lines Tg5516 and Tg5519 that express huRANKL at a physiological relevant tissue-specific pattern. Tg5516 mice expressing huRANKL at low levels develop mild trabecular bone loss, while a more severe osteoporotic phenotype is identified in the Tg5519 line overexpressing huRANKL with features of severe trabecular bone loss and cortical porosity (23). Simultaneous overexpression of RANKL and TNF in Tg197/TgRANKL mice resulted in an aggressive arthritic phenotype, characterized by earlier arthritis onset and exacerbated clinical symptoms, such as reduced body weight gain and increased arthritis scores compared to Tg197 arthritic control mice (Figures 2A,B). Histopathological analysis at 6 weeks of age, when arthritic manifestations in Tg197 mice were restricted on synovial hyperplasia and focal pannus formation, showed significantly increased arthritis progression in Tg197/Tg5519 mice characterized by aggravated inflammatory pannus formation, increased osteoclastogenesis, massive bone destruction and surface cartilage degradation as indicated by staining with hematoxylin/eosin, TRAP and Toluidine blue (Figures 2C,D). Similarly, Tg197/Tg5516 mice displayed an exacerbation of inflammatory arthritis compared to Tg197 mice but to a lesser extent as regards Tg197/Tg5519 mice, indicating a RANKL dose effect on arthritis progression (Figures 2C,D).

**Figure 3:**
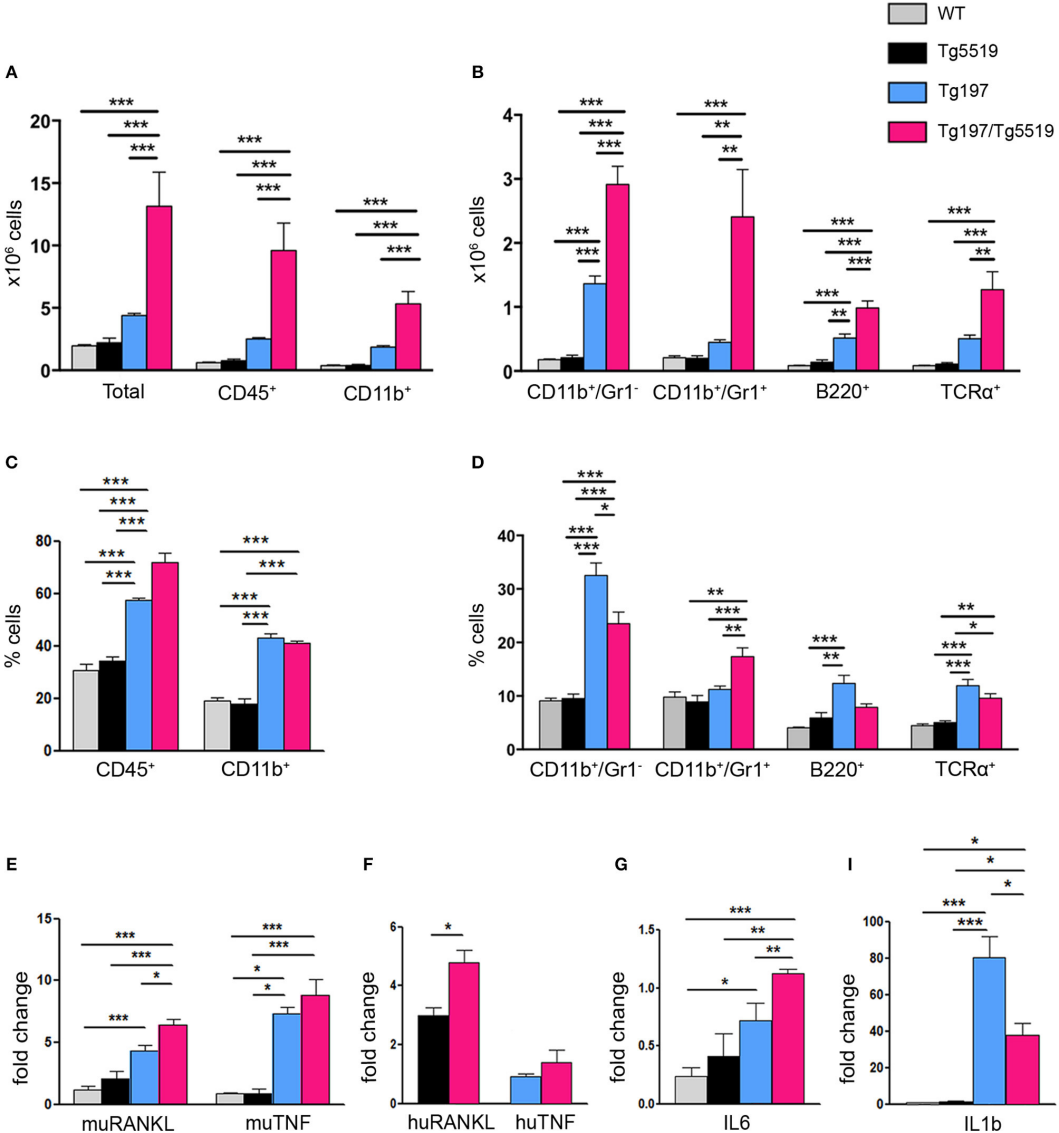
Increased inflammatory cell infiltration in the synovium of Tg197/TgRANKL mice. **(A,B)** Absolute cell counts and **(C,D)** percentage of synovial subpopulations from 6 week-old Tg197/Tg5519 mice and sex-matched littermates (WT, Tg5519, Tg197) as determined by flow cytometry using antibodies against CD45 (hematopoietic cells), CD11b (myeloid cells), Gr1 (granulocytes), B220 (B lymphocytes) and TCRα (T lymphocytes) (*n* = 4–5 per genotype). qPCR analysis in inflamed ankles from 6 week-old Tg197/Tg5519 mice and littermate controls (*n* = 3–4) for **(E)** mouse RANKL and mouse TNF, **(F)** human RANKL and human TNF, **(G)** IL6 and **(I)** IL1b cytokine. Data represent mean values ± SEM. One-Way ANOVA and Tukey *post-hoc* test was performed for more than two groups and Student's *t*-test for two groups. Asterisks mark statistically significant difference (^*^p < 0.05, ^**^p < 0.01, ^***^p < 0.001).

A hallmark of RA is the accumulation of inflammatory cells such as monocytes, neutrophils and lymphocytes in the proliferating synovium that penetrates the cartilage and the bone in the form of pannus causing aberrant joint destruction. So far, the role of RANKL in inflammation remains enigmatic. To examine whether the exacerbated arthritis phenotype developed in Tg197/TgRANKL mice correlates with an increased inflammatory profile, we analyzed the synovial tissue for inflammatory cells through flow cytometry (Figure 3). Our analysis showed a significant increase in the number of infiltrated cells extracted from Tg197/Tg5519 inflamed ankles compared to Tg197 mice (Tg197/Tg5519: 13.15 ± 2.72 × 10^6^ cells vs. Tg197: 4.39 ± 0.16 × 10^6^ cells), supporting arthritis exacerbation. More specifically, CD45^+^ hematopoietic-derived cell infiltrates were increased 4 times in the synovium of Tg197/Tg5519 mice compared to Tg197 mice (Tg197/Tg5519: 9.59 ± 2.2 × 10^6^ cells vs. Tg197: 2.51 ± 0.09 × 10^6^ cells), while no statistical changes were identified between Tg5519 and WT mice (Figure 3A). The synovium of Tg197/Tg5519 mice was infiltrated by 2-fold more CD11b^+^Gr1^−^ monocytes/macrophages (Tg197/Tg5519: 2.91 ± 0.28 × 10^6^ cells vs. Tg197: 1.36 ± 0.11 × 10^6^ cells), and 5-fold more CD11b^+^Gr1^+^ granulocytes (Tg197/Tg5519: 2.4 ± 0.73 × 10^6^ cells vs. Tg197: 0.44 ± 0.04 × 10^6^ cells) than Tg197 mice, where monocytes and synovial macrophages are the dominant inflammatory cells (Figure 3B). The absolute numbers of B220+ B lymphocytes (Tg197/Tg5519: 0.98 ± 0.11 × 10^6^ cells vs. Tg197: 0.51 ± 0.06 × 10^6^ cells) and TCRα+ T lymphocytes (Tg197/Tg5519: 1.27 ± 0.27 × 10^6^ cells vs. Tg197: 0.5 ± 0.05 × 10^6^ cells) were also significantly increased in comparison to Tg197 mice, however to a lesser extent than myeloid cells (Figure 3B).

As regards the percentage of inflammatory cells in the arthritic synovium, flow cytometry revealed a statistical increase of the percentage of CD45^+^ hematopoietic cells in Tg197/Tg5519 mice compared to Tg197, supporting exacerbation of inflammation (Figure 3C). Even though the percentages of B and T lymphocytes were rather low in the inflamed synovium of double transgenic mice (Tg197/Tg5519) and arthritic mice (Tg197), CD11b^+^/Gr1^−^ macrophages/monocytes were prevalent in Tg197, while CD11b^+^/Gr1^+^ granulocytes in Tg197/Tg5519 mice (Figure 3D), indicating probable differences in pathogenic mechanisms. Similarly, inflamed synovium from Tg197/Tg5516 mice also contained increased numbers and percentages of CD45^+^ hematopoietic cells (Tg197/Tg5516: 72.4 ± 1.2% cells vs. Tg197: 59.02 ± 0.37%) and CD11b^+^/Gr1^+^ granulocytes (Tg197/Tg5516: 15.63 ± 0.62% vs. Tg197: 10.9 ± 1%) compared to Tg197. Collectively, the aberrant co-expression of TNF and RANKL, modifies the inflammatory profile in the inflamed ankles toward a massive accumulation of inflammatory cells mainly of myeloid origin.

Furthermore, the cytokine profile of the inflamed ankle joints was investigated through qPCR. Expression analysis for endogenous RANKL showed a progressive increase in Tg197 and Tg197/Tg5519 mice compared to control groups WT and Tg5519, indicating a positive correlation with arthritis severity (Figure 3E). Similarly, the expression levels of the huRANKL transgene were significantly increased in Tg197/Tg5519 mice compared to Tg5519 mice (Figure 3F), supporting an impact of the arthritic milieu in the regulation of the transgene's expression since it carries regulatory regions. In contrast, the expression levels of the endogenous TNF and those of the human TNF transgene were similar between Tg197 and Tg197/Tg5519 mice (Figures 3E,F), excluding their possible involvement in arthritis aggravation upon RANKL overexpression. We also investigated the expression of two proinflammatory cytokines, IL-6 and IL-1b in inflamed ankles (Figures 3G–I). Both cytokines were significantly upregulated in Tg197 mice compared to WT mice. The expression level of IL-6, a proinflammatory cytokine of the acute phase response that promotes neutrophil production, was further 1.5-fold increased in Tg197/Tg5519 mice compared to Tg197 (Figure 3G), in line with the granulocytic arthritic phenotype developed in such mice (Figures 3B,D). Instead, IL-1b, a proinflammatory cytokine expressed by activated macrophages, was 2-fold decreased in Tg197/Tg5519 compared to Tg197 mice (Figure 3I), which could be explained by the proportional decrease of macrophages in the inflamed synovium of Tg197/Tg5519 mice (Figure 3D).

### Cooperative Effect of RANKL and TNF in Local and Systemic Bone Resorption

**Figure 4:**
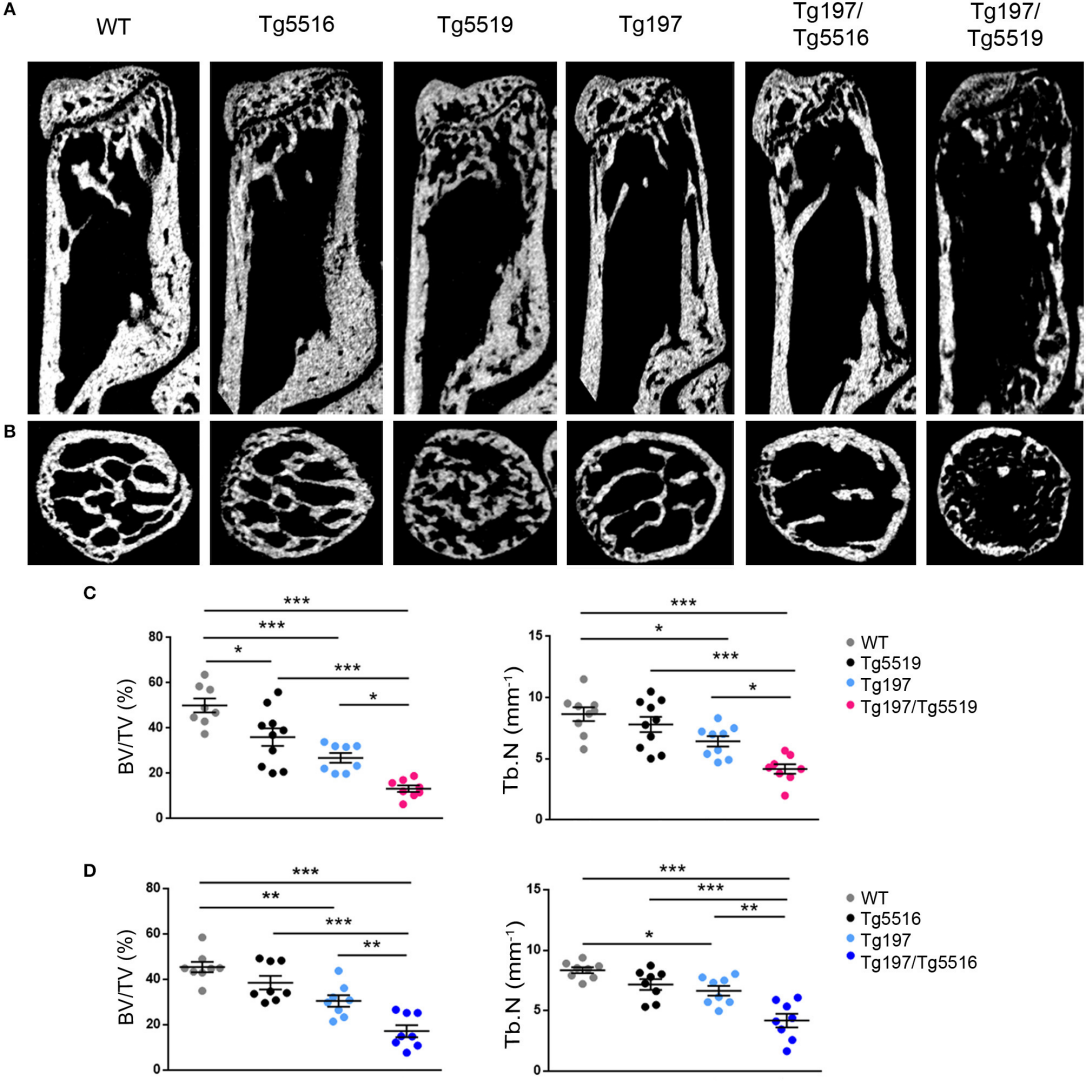
Synergistic effect of RANKL and TNF in inflamed bone loss. MicroCT analysis in the trabecular region of the calcaneous bone from mice of each group at 6 weeks of age as shown by **(A)** representative longitudinal sections and **(B)** cross-sectional sections. Quantitative measurements with microCT in the trabecular area of the calcaneous bone for BV/TV (Bone Volume/Total Volume, %), and Tb.N (Trabecular Number per mm) in **(C)** Tg197/Tg5519 and littermates (*n* = 8–10, equal number of sexes per genotype), and **(D)** Tg197/Tg5516 and littermates (*n* = 8, equal number of sexes per genotype). Data represent mean values ± SEM. One-Way ANOVA and Tukey *post-hoc* test was performed for more than two groups. Asterisks mark statistically significant difference (^*^p < 0.05, ^**^p < 0.01, ^***^p < 0.001).

We further investigated whether the exacerbated arthritis phenotype identified in Tg197/TgRANKL mice affected bone erosion locally. Histological examination of the inflamed ankles from Tg197/TgRANKL mice showed pronounced inflammatory bone destruction. To quantify bone loss locally, we performed microcomputed tomography (microCT) at the trabecular region of the calcaneous bone, which is proximal to the inflamed synovium and contains an organized trabecular structure. Both TgRANKL osteoporotic mice and Tg197 arthritic mice showed trabecular bone loss in the calcaneous bone at 6 weeks of age (Figure 4), while the calcaneous bone loss was further exacerbated in Tg197/RANKL mice compared to the control littermate groups (WT, TgRANKL, Tg197). In the severe osteoporotic model Tg5519 the presence of the huTNF transgene promoted bone loss in an additive manner. Assessment of the trabecular bone volume fraction (BV/TV, %) demonstrated a 28% reduction in Tg5519, 46% in Tg197 and 73% in Tg197/Tg5519 compared to WT group (Figures 4A–C). Furthermore, a synergistic effect was identified when huTNF was introduced in the mild osteoporosis model Tg5516, as Tg197/Tg5516 mice displayed a 62% reduction in BV/TV, while Tg5516 and Tg197 together reached a 48% reduction compared to WT (Figure 4D). These results indicate that the exacerbated arthritis developed in Tg197/TgRANKL mice coincides with a cooperative local bone loss.

**Figure 5:**
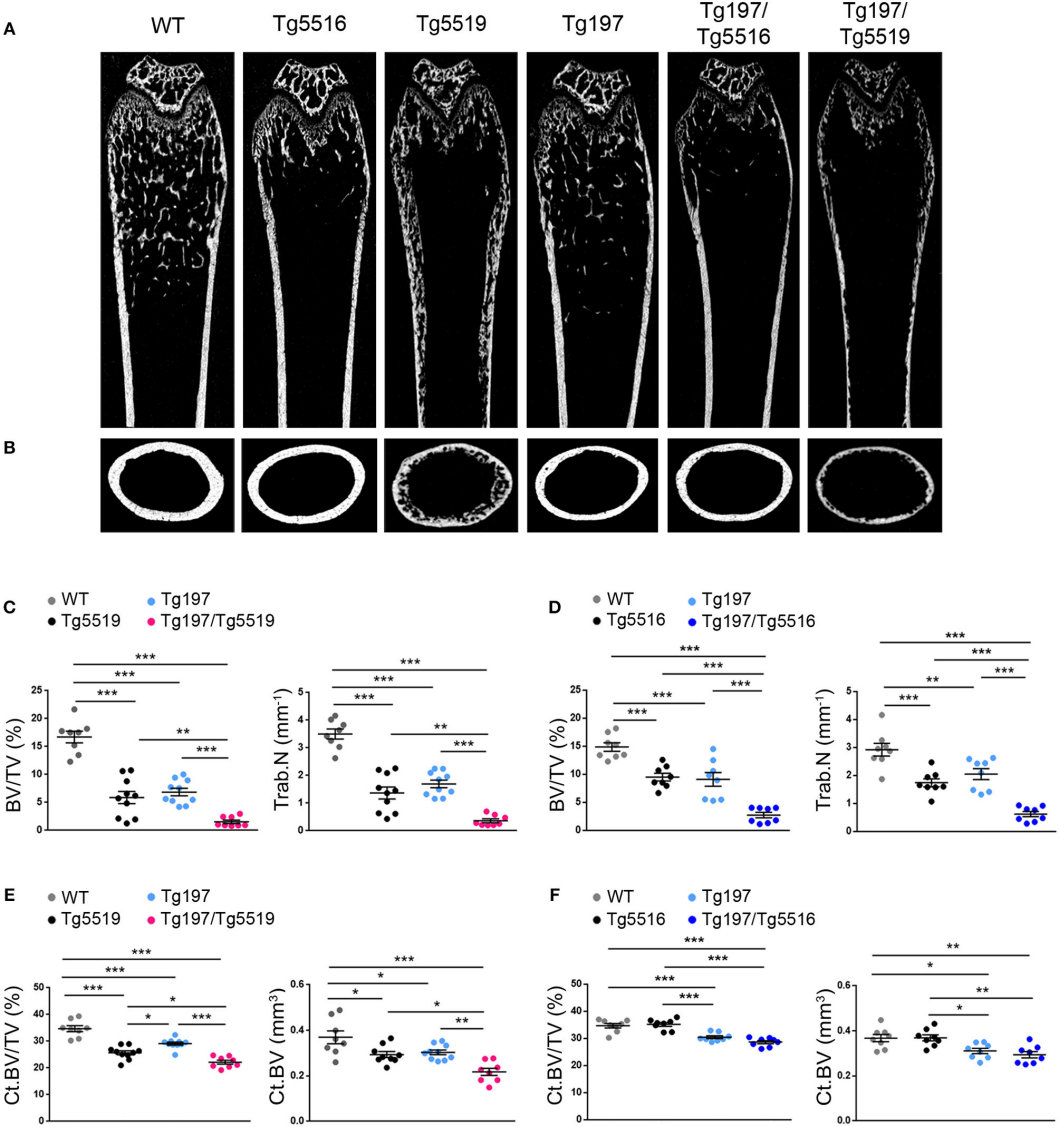
Cooperative effect of RANKL and TNF in systemic bone loss. MicroCT analysis in the distal femur from mice of each genotype at 6 weeks of age as shown by **(A)** representative longitudinal sections and **(B)** cross-sectional sections at mid-diaphysis. Quantitative analysis with microCT of trabecular bone in the metaphyseal region of the distal femur of **(C)** Tg197/Tg5519 mice and their littermate controls (*n* = 8–10, equal number of sexes per genotype) and **(D)** Tg197/Tg5516 mice and their littermate controls (*n* = 8, equal number of sexes per genotype) for BV/TV (Bone Volume/Total Volume, %), and Tb.N (Trabecular Number per mm). Quantitative analysis with microCT of cortical bone in the mid-diaphyseal region of the femur of **(E)** Tg197/Tg5519 mice compared to their littermate controls (*n* = 8–10, equal number of sexes per genotype), and **(F)** Tg197/Tg5516 and their littermates (*n* = 8, equal number of sexes per genotype) for Ct.BV/TV (Cortical Bone Volume/Total Volume, %), and Ct. BV (Cortical Bone Volume, mm^3^). Data represent mean values ± SEM. One-Way ANOVA and Tukey *post-hoc* test was performed for more than two groups. Asterisks mark statistically significant difference (^*^p < 0.05, ^**^p < 0.01, ^***^p < 0.001).

To investigate whether simultaneous overexpression of RANKL and TNF also affected other skeletal sites outside of the inflamed ankles, we analyzed both metaphyseal and diaphyseal regions in distal femurs from Tg197, TgRANKL, and Tg197/TgRANKL mice (Figure 5). Similarly to the calcaneous bone, both the trabecular and the cortical regions of the non-inflamed Tg197/TgRANKL femurs displayed exacerbated bone loss. The severe osteoporotic phenotype in Tg5519 was further aggravated, while mild osteoporosis in Tg5516 mice converted to severe osteoporosis upon huTNF expression, indicating that TNF and RANKL also cooperate in systemic bone loss.

### Proteomic Analysis of Inflamed Joints

**Figure 6:**
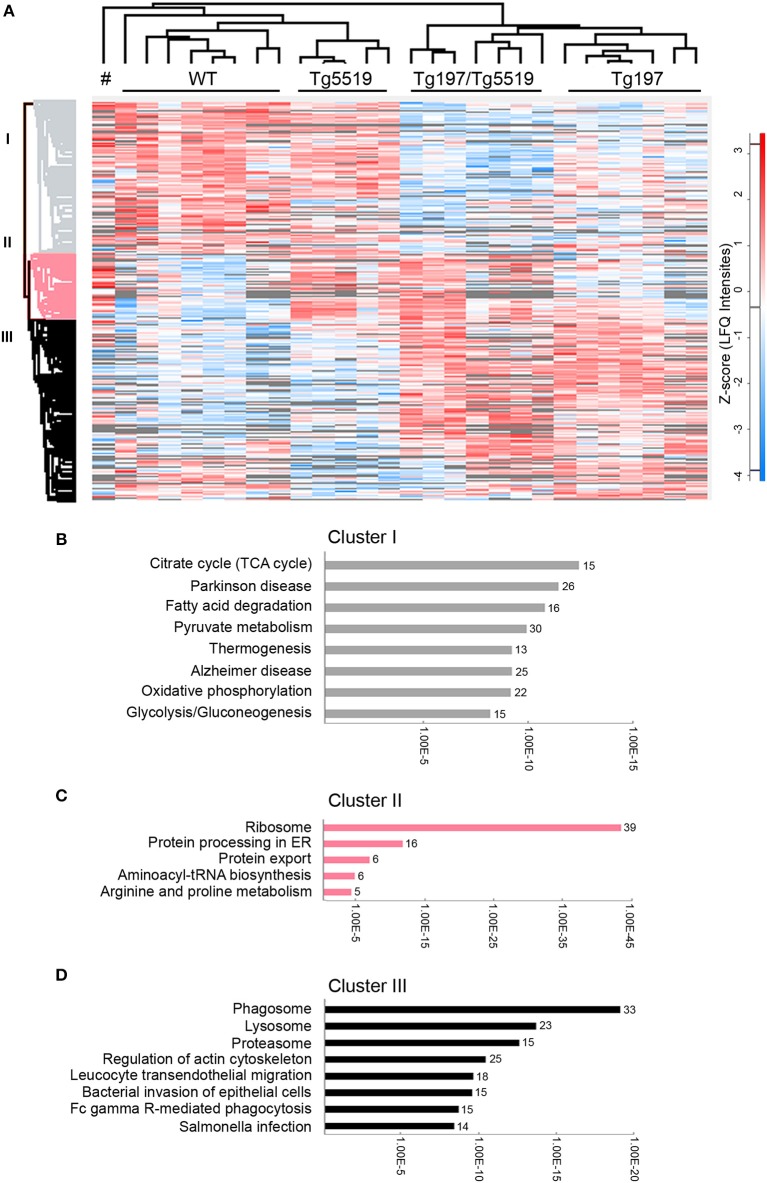
Proteomic analysis in inflamed ankle joints of Tg197/TgRANKL mice. Comparative proteomic analysis using LC-MS/MS and label free quantitation in ankles from 6 week-old Tg197/Tg5519 mice and control groups including Tg197, Tg5519, and WT littermate mice (*n* = 6–8 biological replicas). **(A)** Heat map of statistical significant proteins (one-way ANOVA analysis). Columns represent each individual sample, labeled on top, and each row represents single proteins with an assigned color from blue (low expression) to red (high expression). Not detectable proteins are colored gray. Hierarchical Euclidean clustering created 3 protein clusters (gray, pink, and black). # indicates one Tg5519 mouse. Annotation enrichment analysis was performed using KEGG pathways database for **(B)** cluster I, **(C)** cluster II, and **(D)** cluster III.

To identify altered biological processes and changes in the proteome at osteolytic inflammatory arthritis aggravated by the overexpression of RANKL, we utilized a comparative proteomic approach using LC-MS/MS and label free quantitation in ankles from 6 week-old Tg197/Tg5519 transgenic mice and control groups, including Tg197, Tg5519, and WT littermate mice. Analysis was performed on whole ankle joints in order to capture deregulated protein networks at the time of isolation while also maintaining all the populations and the potential interactions in inflamed ankles. For each ankle we quantified 2,009 proteins using label-free quantitation (LFQ) determined by the MaxQuant software (31, 32). We achieved high biological reproducibility as reflected by the unsupervised clustering of the genotypes in the composed heatmap (Figure 6A). To define significant regulated proteins, we performed one-way ANOVA analysis between the four genotypes and identified 1,019 significantly regulated proteins (Figure 6A). Hierarchical clustering of significantly regulated proteins revealed three major groups. Cluster I consists of 403 proteins, Cluster II of 179 and Cluster III of 437 proteins (Figure 6A). Bioinformatic “annotation enrichment analysis” in these clusters using ClueGO/CluePedia software (29) identified the main biological pathways (KEGG database) regulated by these proteins. Cluster I contained proteins involved in basic metabolic processes such as citrate cycle (TCA), oxidative phosphorylation or glycolysis/gluconeogenesis that were found specifically downregulated in arthritic groups Tg197 and Tg197Tg5519 (Figure 6B and Supplementary Table 1). Cluster II is composed mostly of ribosomal proteins which are enriched in huRANKL overexpressing mice Tg5519 and Tg197/Tg5519 (Figure 6C and Supplementary Table 2). Enrichment analysis in Cluster III revealed a high prevalence of proteins involved in phagosome, lysosome, proteasome, cytoskeleton regulation, leukocyte transendothelial migration and Fcγ-receptor-mediated phagocytosis in arthritic Tg197 and Tg197/Tg5519 mice compared to control groups Tg5519 and WT (Figure 6D and Supplementary Table 3), suggesting activation of immune responses.

**Figure 7:**
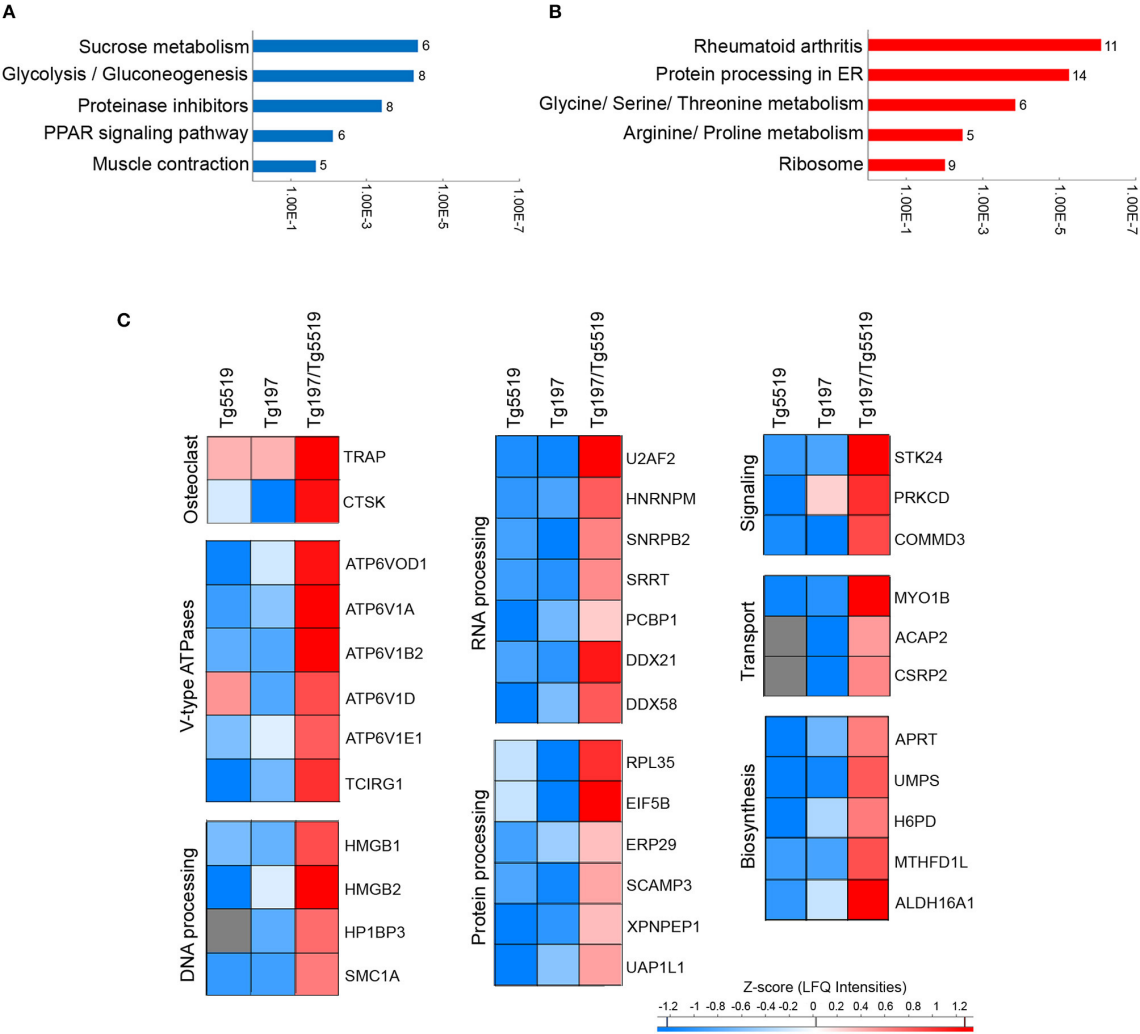
Identification of upregulated proteins in the inflamed ankles of Tg197/TgRANKL mice. Enrichment annotation analysis based on KEGG database for **(A)** downregulated and **(B)** upregulated proteins in Tg197/Tg5519 compared to Tg197 mice. Bars represent the –Log(*p*-value) of pathways and the associated number of genes is presented at the end of the bar. **(C)** Abundance heat maps for proteins upregulated in Tg197/Tg5519 mice compared to Tg197 and Tg5519 control mice (Tukey's analysis). The average abundance of biological replicas (*n* = 6–8) is represented in each cell of the heat map.

To elucidate the most prominent proteins involved in RA aggravation by RANKL, Tukey's honestly significant difference was performed on the ANOVA significant hits. A total of 231 proteins, 120 downregulated and 111 upregulated, were found statistically altered in Tg197/Tg5519 compared to Tg197 mice. Enrichment analysis in downregulated proteins based on KEGG pathway database revealed classification to processes related with metabolism, and muscle contraction (Figure 7A and Supplementary Table 4). In contrast, the upregulated proteins were grouped to processes associated with RA, protein processing and amino acid metabolism (Figure 7B and Supplementary Table 5).

To exclude a possible involvement of the osteoporotic background in the deregulated proteins identified in Tg197/Tg5519 compared to Tg197, we examined which of the above mentioned 231 differentially expressed proteins were also statistically significant between Tg197/Tg5519 and Tg5519 mice. This analysis revealed 65 proteins downregulated (Supplementary Table 6) and 36 upregulated (Figure 7C, Table 1) in Tg197/Tg5519 compared to control groups Tg197 and Tg5519. Subcategorization of the 65 significantly downregulated proteins in Tg197/Tg5519 mice based on their biological function, showed that the majority of the proteins participated in metabolic processes of carbohydrates, lipids, amino acids and nucleotides as well as in mitochondrial function (Supplementary Table 6). On the other hand, the proteome of the inflamed ankles from Tg197/Tg5519 mice was enriched for proteins expressed in activated osteoclasts and vacuolar-type H+ ATPase subunits either osteoclast-specific or ubiquitous (Figure 7C, Table 1), indicating extended bone resorption. Similarly, upregulation was observed for proteins involved in DNA, RNA and protein processing, suggesting activation of chromatin remodeling and gene expression. Moreover, increased levels have been identified for proteins involved in intracellular signal transduction, vacuolar transport and cell migration. Many of the upregulated proteins have been implicated in inflammatory responses, and cell proliferation regulation that fully correlate with the aggressive inflammatory phenotype developed in the ankles of Tg197/Tg5519 mice.

**Table 1:** Proteins identified significantly overexpressed in the ankles from Tg197/Tg5519 mice compared to those isolated from their littermates Tg197, and Tg5519.

**Gene Name**	**Protein ID**	**ANOVA *p*-value**	**WT**	**Tg5519**	**Tg197**	**Tg197/Tg5519**
**OSTEOCLAST ACTIVITY**						
Acp5 (Trap)	Q05117	5.1E-08	18.46	21.24	21.24	22.73
Ctsk	P55097	2.2E-04	0.00	19.14	17.94	20.61
**V-TYPE ATPASES**						
Atp6v0d1	P51863	8.8E-07	18.26	19.14	19.52	20.05
Atp6v1a	P50516	7.0E-10	21.83	22.60	22.74	23.37
Atp6v1b2	P62814	1.2E-10	21.20	22.03	22.01	22.73
Atp6v1d	P57746	1.9E-08	17.95	19.33	18.78	19.47
Atp6v1e1	P50518	1.5E-08	20.34	21.33	21.50	21.85
Tcirg1	Q9JHF5	2.2E-08	18.13	19.23	19.59	20.57
**DNA PROCESSING**						
Hmgb1	P63158	9.5E-03	21.69	21.06	20.96	22.48
Hmgb2	P30681	8.3E-05	20.44	19.78	20.59	21.66
Hp1bp3	Q3TEA8	1.2E-03	17.51	0.00	17.47	18.22
Smc1a	Q9CU62	2.6E-02	17.38	17.11	17.13	17.75
**RNA PROCESSING**						
U2af2	P26369	2.7E-04	19.10	18.86	18.83	19.91
Hnrnpm	Q9D0E1	2.5E-03	21.95	22.03	22.06	22.46
Snrpd2	Q9CQI7	1.3E-02	19.58	19.11	18.96	19.68
Srrt	Q99MR6	5.3E-03	17.36	17.31	17.25	18.03
Pcbp1	P60335	1.3E-03	22.19	21.93	22.11	22.39
Ddx21	Q9JIK5	9.7E-04	18.70	18.82	18.76	19.64
Ddx58	Q6Q899	1.5E-03	17.00	17.40	17.77	18.56
**PROTEIN PROCESSING**						
Rpl35	Q6ZWV7	3.7E-03	20.45	21.16	20.43	22.08
Eif5b	Q05D44	9.0E-06	17.17	17.64	17.03	18.64
Erp29	P57759	7.5E-05	20.24	20.59	20.77	21.09
Scamp3	O35609	1.7E-03	18.44	18.62	18.54	18.95
Xpnpep1	Q6P1B1	9.1E-03	20.79	20.63	20.68	20.98
Uap1l1	Q3TW96	1.4E-05	20.54	20.69	20.96	21.34
**SIGNAL TRANSDUCTION**						
Stk24	Q99KH8	5.9E-04	17.66	17.96	18.02	19.43
Prkcd	P28867	7.6E-06	17.96	18.36	19.31	19.83
Commd3	Q63829	1.8E-02	0.00	17.98	17.91	18.70
**VACUOLAR TRANSPORT, ENDOCYTOSIS, INVASIVENESS**						
Myo1b	P46735	2.2E-03	17.84	18.23	18.27	19.08
Acap2	Q6ZQK5	7.8E-03	17.72	0.00	17.82	18.36
Csrp2	P97314	5.1E-05	18.22	0.00	18.37	19.36
**BIOSYNTHETIC PROCESS**						
Aprt	P08030	9.7E-06	19.19	19.41	19.72	20.38
Umps	P13439	2.3E-02	0.00	17.31	17.35	18.34
H6pd	Q8CFX1	6.2E-07	18.94	19.07	19.31	19.58
Mthfd1l	Q3V3R1	4.0E-04	19.55	19.73	19.75	20.25
Aldh6a1	Q9EQ20	7.2E-03	22.27	22.04	21.68	21.53

*Logarithmic LFQ mean values are provided for each genotype*.

## Discussion

The importance of the RANKL/RANK/OPG system in the development of bone destruction in RA has been recently established, since RANKL is highly expressed in the synovial tissue of RA patients (33–35) and inhibition of RANKL with denosumab results in amelioration of bone destruction in RA (36, 37). Paradoxically, there are limited clinical trials that inhibit RANKL in RA, and from the available ones an effectiveness has been demonstrated for bone resorption but not for inflammation during a short-term treatment period from 6 to 12 months (36–39). Thus, the role of RANKL in the progression of inflammation in RA remains unclear. Here, we investigated if the progression of TNF-mediated erosive inflammatory arthritis is affected either by genetic inactivation (22) or overexpression of RANKL in transgenic mouse models (23). Our previous studies have shown that the G278R substitution identified in Rankl^tles/tles^ mice allows normal RANKL gene expression and protein production but abrogates RANKL trimer formation and subsequently receptor binding. Therefore, mutant RANKL lacks biological activity as it fails to induce osteoclastogenesis both *ex vivo* and *in vivo* leading to an osteopetrotic phenotype (22). Our results demonstrated that modeled arthritis was significantly attenuated in the absence of functional RANKL, as shown by the absence of clinical arthritis signs and significant decrease in synovial hyperplasia. The unexpected improvement of survival in Tg197/Rankl^tles/tles^ mice compared to control Rankl^tles/tles^ mice, could indicate a compensatory role for TNF in a RANKL-null background and needs further investigation. It is possible that the observed amelioration of arthritis is caused by the osteopetrotic phenotype rather than RANKL inactivation *per se*. In contrast, previous reports using c-fos deficient osteopetrotic mice crossed with TNF arthritic mice demonstrated that osteopetrosis is dispensable for TNF-mediated arthritis as synovial inflammation was not affected whereas bone resorption was blocked (40), supporting RANKL involvement in arthritis as shown in Tg197/Rankl^tles/tles^ mice. Moreover, it is also possible that the attenuation of arthritis identified in Tg197/Rankl^tles/tles^ mice is caused by the failure of RANKL deficient mice to develop a functional immune system (5, 6, 22).

Although several studies have revealed that TNF mediates osteoclastogenesis using *in vitro* cell culture systems (19, 20), there is still a central controversy of whether TNF can compensate for RANKL during osteoclastogenesis *in vivo*. Even though administration of high doses of exogenous TNF leads to the formation of osteoclast-like cells in RANK knockout mice at the site of calvarial injection (41), introduction of the Tg3647 TNF-expressing transgenic model displaying late onset arthritis in a RANK knockout background, showed that upon TNF overexpression osteoclastogenesis does not occur in the absence of RANKL/RANK signaling (42). Our results demonstrated that TNF overexpression could not compensate for RANKL-mediated osteopetrosis in Tg197/Rankl^tles/tles^ mice, supported by the absence of osteoclasts in the bone marrow compartment. The fact that osteoclasts were identified between the pannus and bone interface in Tg197/Rankl^tles/tles^ mice, indicates that this effect is driven by TNF-induced inflammation *in vivo*. However, the involvement of a subtle RANKL signaling in TNF-driven osteoclastogenesis cannot be excluded and needs further investigation. Similarly, previous reports have shown that induction of K/BxN serum transfer arthritis in RANK-deleted mice, resulted in osteoclastogenesis in the inflamed synovium but not in the bone marrow, supporting RANKL-indepedent mechanisms for osteoclast formation *in vivo* in a sufficiently inflamed environment (43).

Following a similar approach, the effect of RANKL overexpression in arthritis progression was studied in Tg197/TgRANKL double transgenic mice that simultaneously overexpress TNF and RANKL. Our results demonstrated that abundance of RANKL accelerated TNF-driven arthritis onset and disease severity characterized by massive osteoclastogenesis and bone resorption, aggressive pannus expansion and immense infiltration of inflammatory cells mainly of myeloid origin. Even though in the inflamed ankles of Tg197 mice the dominant inflammatory cells were CD11b^+^Gr1^−^ monocytes and synovial macrophages, the synovium of Tg197/Tg5519 mice had a 5-fold increase in CD11b^+^Gr1^+^ granulocytes and 2-fold in CD11b^+^Gr1^−^ monocytes/macrophages. The percent composition of various infiltrated populations showed a clear prevalence of granulocytes in TNF-driven arthritis upon RANKL overexpression. Neutrophils, the most abundant type of granulocytes, are short-lived and highly motile cells that constitute an essential component in innate immune system, as they are among the first cells that arrive in inflamed tissues (44). They are involved in various chronic inflammatory diseases such as RA, where are found in synovial fluid and rheumatoid pannus. It has been previously demonstrated that the membrane-associated form of RANKL is expressed in healthy blood neutrophils as well as in SF neutrophils (45), suggesting a role for inflammatory neutrophils infiltrated at the hypertrophied synovium, in osteoclastogenesis and bone resorption. Apart from that, RANKL was recently demonstrated to potently activate human neutrophil degranulation (46) and treatment with anti-RANKL improved cardiac infarct size and function by potentially impacting on neutrophil-mediated injury and repair (47). Thus, the dramatic increase in the population of granulocytes in inflamed ankles from Tg197/Tg5519 mice could promote bone destruction.

Proteomics, the largescale study of the proteome, has emerged as a powerful technique to identify biomarkers for diagnosis, prognosis, disease monitoring and discovery of novel disease targets in RA (48). To identify proteome alterations in osteolytic inflammatory arthritis aggravated by the overexpression of RANKL, we utilized a comparative proteomic approach in inflamed ankles from Tg197/Tg5519 and control mice. Our analysis revealed 65 significantly downregulated proteins in Tg197/Tg5519 mice compared to Tg197 and Tg5519 mice, while their classification based on biological function, showed that the majority of the proteins participated in metabolic processes of carbohydrates, lipids, amino acids and nucleotides as well as in mitochondrial function. These results indicate that severe inflammation developed in the ankles of Tg197/Tg5519 mice is related to altered metabolic profile and probably mitochondria dysfunction as many mitochondrial proteins were downregulated (Supplementary Table 6). In RA the inflamed joint is profoundly hypoxic as a result of dysregulated angiogenesis, impaired mitochondrial function and inflammation, which leads to a bioenergetic crisis. Under these conditions synovial cells display adaptive survival responses, which in conjunction with altered metabolism, activate key transcriptional signaling pathways that further exacerbate inflammation (49). Notably, there is also downregulation of proteins functioning as protease inhibitors, such as Alpha-1-antitrypsin encoded by the *SERPINA1* gene, that protect tissues from enzymes of inflammatory cells, especially neutrophil elastase (50), suggesting extensive tissue damage in Tg197/Tg5519 mice. Moreover, downregulation of proteins involved in muscle contraction in Tg197/Tg5519 mice is indicative of muscle degeneration caused by movement impairment due to severe arthritis progression.

In contrast, the proteome of the inflamed ankles from Tg197/Tg5519 mice was enriched for proteins expressed in activated osteoclasts, including TRAP and cathepsin K (CTSK), and vacuolar-type H+ ATPase subunits. TRAP prompts the dephosphorylation of bone matrix phosphoproteins and allows osteoclast migration, and further resorption to occur (51), while Cathepsin K, a member of cysteine proteases, is involved in the degradation of bone matrix proteins, especially type I collagen (52). Apart from bone resorption, Cathepsin K plays an important role in the immune system as shown by suppression of experimental arthritis through its pharmacological inhibition (53). The vacuolar type H+ ATPases (V-ATPase) are ATP-driven proton pumps that establish and maintain the acidic environment of intracellular organelles, including secretory granules, endosomes, and lysosomes, as well as extracellular compartments by specialized cells (54). Within intracellular membranes, V-ATPases function in a variety of processes, including antigen processing in dendritic cells and lysosomal degradation, while their presence in the plasma membrane mediates extracellular acidification (55). The mammalian V-ATPase proton pump is a macromolecular complex composed of at least 14 subunits that are expressed and function in a tissue-specific manner. Genetic studies implicate a critical role for subunits ATP6V1B2, ATP6V1C1, ATPV0D2, and ATP6V0A3 (TCIRG1) in osteoclast activity as relevant mutations lead to osteopetrosis (56). Osteoclasts employ plasma membrane V-ATPases to release hydrogen ions (H+) into the resorption lacunae in order to dissolve the mineral component of bone and concomitantly to enhance the activity of enzymes that digest the organic matrix (56). The fact that Tg197/Tg5519 inflamed ankles overexpress various V-ATPase subunits either osteoclast specific such as ATP6V1B2, and TCIRG1 or ubiquitous ATP6V1A, ATP6V1E1, and ATPV0D1 indicates an overwhelming osteoclastic activity that causes massive joint destruction, which is also confirmed by the histological analysis. Upregulated V-ATPase subunits could also have an impact on inflammatory responses such as phagocytosis, cytokine secretion and exocytosis of neutrophil granules (57, 58). Notably, recent studies have shown that in inflammatory conditions, osteoclasts can differentiate from dendritic cells in the presence of RANKL and behave as antigen-presenting cells (59). Therefore, increased osteoclastogenesis identified in Tg197/TgRANKL mice could not only contribute to bone destruction, but may also participate in perpetuating the inflammatory response.

In inflamed ankles from Tg197/Tg5519 mice there is also upregulated expression of proteins involved in DNA, RNA and protein processing, suggesting activation of chromatin remodeling and gene expression. Of special importance are DNA binding proteins, HMGB1 and HMGB2, members of the High-mobility group box (HMGB) family displaying two functions. In the nucleus, HMGB proteins bind to DNA in a DNA structure-dependent but nucleotide sequence-independent manner to function in chromatin remodeling. Extracellularly, HMGB proteins function as alarmins or damage-associated molecular pattern (DAMP) molecules, which are endogenous molecules released upon tissue damage to activate the immune system and drive inflammatory responses (60). Circulating HMGB1, the prototype member, has a crucial role in sterile inflammation caused by tissue injury or mitochondria damage, while its levels are increased in many human inflammatory diseases such as rheumatoid arthritis and their associated experimental models (61–63). Secreted HMGB1 binds to several immune receptors, principally toll-like receptors (TLRs) and through activation of NF-κB signaling (64) triggers inflammation by inducing cytokine release and recruitment of leucocytes. Thus, upregulation of HMGB1 and HMGB2 in inflamed ankles suggests extensive tissue damage and sustained inflammatory responses.

Moreover, proteomic analysis in Tg197/Tg5519 inflamed ankles identified high expression of RNA-binding proteins involved in mRNA splicing, and miRNA biogenesis. Among these proteins, U2AF2 (U2 Small Nuclear RNA Auxiliary Factor 2) is a central splicing complex member involved in pre-mRNA splicing and 3′-end processing (65) with an impact in the regulation of transcriptome in activated CD4 T lymphocytes (66). Moreover, HNRNPM (Heterogeneous nuclear ribonucleoprotein M), a component of the spliceosome machinery, promotes alternative spicing, cell proliferation and progression of breast cancer (67), while SRRT (Serrate, RNA Effector Molecule) participates to mRNA splicing and primary miRNA processing (68), it is involved in cell cycle progression at S phase, and its genetic deletion resulted in defective hematopoiesis in bone marrow and thymus (69). DDX21 and DDX58, as RNA helicases unwind their RNA substrates, and are involved in multiple biological processes related to RNA metabolism, including viral dsRNA sensing by innate cells, initiation of host antiviral responses and production of proinflammatory cytokines (70). Emerging evidence indicate that HMGBs bind to immunogenic nucleic acids (promiscuous sensing), which is required for subsequent recognition by specific pattern recognition receptors (discriminative sensing) such as DDXs to activate the innate immune responses. Such helicases also interact with endogenous RNAs regulating ribosome biogenesis (71) or translation of specific targets such as NF-κB1 (72). This category of nuclear RNA-binding proteins suggests increased transcription, RNA biogenesis and processing, while it remains unclear whether there is a specific correlation with regulation of inflammatory genes.

As regards intracellular signal transduction, there is abundance of protein kinases such as Serine/threonine kinase (STK24), and Protein kinase Cδ (PKCδ) in Tg197/T5519 ankles. STK24 plays an important role in controlling interleukin 17 (IL-17)-triggered inflammation and autoimmune diseases, since STK24 deficiency or knockdown markedly inhibited IL-17-induced phosphorylation of NF-κB and impaired IL-17-induced chemokines and cytokines expression (73). PKCδ, a signaling kinase with multiple downstream target proteins, is an essential regulator of peripheral B-cell development with a critical role in immune homeostasis. Among its main roles, PKCδ is responsible for the regulation of survival, proliferation, and apoptosis in a variety of cells including lymphocytes, while deficiency in PKCδ leads to systemic autoimmunity (74). Moreover, COMM domain-containing protein 3 (COMMD3) is an uncharacterised member of the COMMD family of proteins that interact with NF-κB and modulate its response (75).

Another group of proteins found upregulated in Tg197/Tg5519 ankles are involved in intracellular vesicular transport, endocytosis and invasiveness in extracellular matrix. MYO1B (Myosin IB) along with actin have been implicated in the control of secretory granule biogenesis and invagination of the plasma membrane during endocytosis (76). ACAP2 (ArfGAP With Coiled-Coil, Ankyrin Repeat And PH Domains 2), is a GTPase-activating protein that plays central role in endocytosis and FcγR-mediated phagocytosis (77), while CRP2 (Cysteine Rich Protein 2) is a new cytoskeletal component of invadopodia promoting breast cancer cell invasiveness and metastasis (78).

To our knowledge, this is the first study showing a proinflammatory role of RANKL in modeled arthritis apart from its well-established bone resorbing properties. A similar effect of RANKL has been identified in experimental periodontitis as RANKL antagonists inhibit both tissue inflammation and bone loss (79). Given that RA is a heterogeneous disease and so far the effect of denosumab in RA has been addressed only for a 12-month period, further studies are needed to investigate the inflammatory properties of RANKL in RA patients. Our results support that RANKL synergizes with TNF not only in local and systemic bone resorption but also in the inflammatory phenotype developed in modeled arthritis. Abundance of RANKL in TNF-driven arthritis worsens arthritis severity as shown by an increase in bone resorption, inflammatory cells and protein biomarkers indicative of extented osteoclastogenesis, tissue damage and activation of the immune system. Moreover, RANKL is essential for physiological and inflammation-induced bone remodeling, while TNF induces osteoclastogenesis *in vivo* at contact sites between synovium and bone. Therefore, RANKL provides an interesting candidate for resolution of inflammatory resorption in RA, whereas a dual inhibition of RANKL and TNF seems a promising therapeutic approach for severe inflammatory osteolytic arthritis.

## Author Contributions

ED conceived and designed the study, supervised experiments, and wrote the manuscript. MP performed and analyzed the majority of experiments and prepared the manuscript. VR performed microCT analysis and edited the manuscript. FV and MS conducted proteomic analysis and edited the manuscript. TT performed statistic analysis in proteomics data. GP provided scientific insight and edited the manuscript.

### Conflict of Interest Statement

The authors declare that the research was conducted in the absence of any commercial or financial relationships that could be construed as a potential conflict of interest.

## References

[1] Fuller K Wong B Fox S Choi Y Chambers TJ. TRANCE is necessary and sufficient for osteoblast-mediated activation of bone resorption in osteoclasts. J Exp Med. (1998) 188:997–1001. 10.1084/jem.188.5.997 9730902 PMC2213394

[2] Lacey DL Timms E Tan HL Kelley MJ Dunstan CR Burgess T. Osteoprotegerin ligand is a cytokine that regulates osteoclast differentiation and activation. Cell (1998) 93:165–76. 10.1016/S0092-8674(00)81569-X 9568710

[3] Yasuda H Shima N Nakagawa N Yamaguchi K Kinosaki M Mochizuki S. Osteoclast differentiation factor is a ligand for osteoprotegerin/osteoclastogenesis-inhibitory factor and is identical to TRANCE/RANKL. Proc Natl Acad Sci USA. (1998) 95:3597–602. 10.1073/pnas.95.7.3597 9520411 PMC19881

[4] Seeman E Delmas PD. Bone quality–the material and structural basis of bone strength and fragility. N Engl J Med. (2006) 354:2250–61. 10.1056/NEJMra053077 16723616

[5] Kong YY Yoshida H Sarosi I Tan HL Timms E Capparelli C. OPGL is a key regulator of osteoclastogenesis, lymphocyte development and lymph-node organogenesis. Nature (1999) 397:315–23. 10.1038/16852 9950424

[6] Kim D Mebius RE MacMicking JD Jung S Cupedo T Castellanos Y Rho J. Regulation of peripheral lymph node genesis by the tumor necrosis factor family member TRANCE. J Exp Med. (2000) 192:1467–78. 10.1084/jem.192.10.1467 11085748 PMC2193182

[7] Dougall WC Glaccum M Charrier K Rohrbach K Brasel K De Smedt T. RANK is essential for osteoclast and lymph node development. Genes Dev. (1999) 13:2412–24. 10.1101/gad.13.18.2412 10500098 PMC317030

[8] Simonet WS Lacey DL Dunstan CR Kelley M Chang MS Lüthy R. Osteoprotegerin: a novel secreted protein involved in the regulation of bone density. Cell (1997) 89:309–19. 10.1016/S0092-8674(00)80209-3 9108485

[9] Darby AJ. Bone formation and resorption in postmenopausal osteoporosis. Lancet (1981) 2:536. 10.1016/S0140-6736(81)90931-4 6115291

[10] Cummings SR San Martin J McClung MR Siris ES Eastell R Reid IR. Denosumab for prevention of fractures in postmenopausal women with osteoporosis. N Engl J Med. (2009) 361:1914. 10.1056/NEJMoa0809493 19671655

[11] Leibbrandt A Penninger JM. Novel functions of RANK(L) signaling in the immune system. Adv Exp Med Biol. (2010) 658:77–94. 10.1007/978-1-4419-1050-9_9 19950018

[12] Tanaka Y. Clinical immunity in bone and joints. J Bone Miner Metab (2018). [Epub ahead of print]. 10.1007/s00774-018-0965-5. 30324535

[13] Firestein GS. Evolving concepts of rheumatoid arthritis. Nature (2003) 423:356–61. 10.1038/nature01661 12748655

[14] Feldmann M Brennan FM Foxwell BM Maini RN. The role of TNF alpha and IL-1 in rheumatoid arthritis. Curr Dir Autoimmun. (2001) 3:188–99. 10.1159/000060522) 11791466

[15] Keffer J Probert L Cazlaris H Georgopoulos S Kaslaris E Kioussis D. Transgenic mice expressing human tumour necrosis factor: a predictive genetic model of arthritis. EMBO J. (1991) 10:4025–31. 10.1002/J.1460-2075.1991.TB04978.X 1721867 PMC453150

[16] Kontoyiannis D Pasparakis M Pizarro TT Cominelli F Kollias G. Impaired on/off regulation of TNF biosynthesis in mice lacking TNF AU- rich elements: implications for joint and gut-associated immunopathologies. Immunity (1999) 10:387–98. 10.1016/S1074-7613(00)80038-2 10204494

[17] Elliott MJ Maini RN Feldmann M Long-Fox A Charles P Bijl H. Repeated therapy with monoclonal antibody to tumour necrosis factor alpha (cA2) in patients with rheumatoid arthritis. Lancet (1994) 344:1125–7. 10.1016/S0140-6736(94)90632-7 7934495

[18] Boyce BF Li P Yao Z Zhang Q Badell IR Schwarz EM. TNF-alpha and pathologic bone resorption. Keio J Med. (2005) 54:127–31. 10.2302/kjm.54.127 16237274

[19] Azuma Y Kaji K Katogi R Takeshita S Kudo A. Tumor necrosis factor-alpha induces differentiation of and bone resorption by osteoclasts. J Biol Chem. (2000) 275:4858–64. 10.1074/jbc.275.7.4858 10671521

[20] Kobayashi K Takahashi N Jimi E Udagawa N Takami M Kotake S. Tumor necrosis factor stimulates osteoclast differentiation by a mechanism independent of the Odf/rankl-rank interaction. J Exp Med. (2000) 191:275–86. 10.1084/jem.191.2.275 10637272 PMC2195746

[21] Fuller K Murphy C Kirstein B Fox SW Chambers TJ. TNFα potently activates osteoclasts, through a direct action independent of and strongly synergistic with RANKL. Endocrinology (2002) 143:1108–18. 10.1210/en.143.3.1108 11861538

[22] Douni E Rinotas V Makrinou E Zwerina J Penninger JM Eliopoulos E. A RANKL G278R mutation causing osteopetrosis identifies a functional amino acid essential for trimer assembly in RANKL and TNF. Hum Mol Genet. (2012) 21:784–98. 10.1093/hmg/ddr510 22068587

[23] Rinotas V Niti A Dacquin R Bonnet N Stolina M Han CY. Novel genetic models of osteoporosis by overexpression of human RANKL in transgenic mice. J Bone Miner Res. (2014) 29:1158–69. 10.1002/jbmr.2112 24127173

[24] Douni E Sfikakis PP Haralambous S Fernandes P Kollias G. Attenuation of inflammatory polyarthritis in TNF transgenic mice by diacerein: comparative analysis with dexamethasone, methotrexate and anti-TNF protocols. Arthritis Res Ther. (2004) 6:R65–72. 10.1186/ar1028 14979939 PMC400419

[25] van 't Hof RJ Rose L Bassonga E Daroszewska A Open source software for semi-automated histomorphometry of bone resorption and formation parameters. Bone (2017) 99:69–79. 10.1016/j.bone.2017.03.051 28366796

[26] Bouxsein ML Boyd SK Christiansen BA Guldberg RE Jepsen KJ Müller R. Guidelines for assessment of bone microstructure in rodents using micro-computed tomography. J Bone Miner Res. (2010) 25:1468–86. 10.1002/jbmr.141 20533309

[27] Armaka M Gkretsi V Kontoyiannis D Kollias G A standardized protocol for the isolation and culture of normal and arthritogenic murine synovial fibroblasts. Protoc Exch (2009). 10.1038/nprot.2009.102

[28] Tyanova S Cox J. Perseus: a bioinformatics platform for integrative analysis of proteomics data in cancer research. Methods Mol Biol. (2018) 1711:133–48. 10.1007/978-1-4939-7493-1_7 29344888

[29] Bindea G Mlecnik B Hackl H Charoentong P Tosolini M Kirilovsky A. ClueGO: a cytoscape plug-in to decipher functionally grouped gene ontology and pathway annotation networks. Bioinformatics (2009) 25:1091–3. 10.1093/bioinformatics/btp101 19237447 PMC2666812

[30] Ogata H Goto S Sato K Fujibuchi W Bono H Kanehisa M. KEGG: kyoto encyclopedia of genes and genomes. Nucleic Acids Res. (1999) 27:29–34. 10.1093/nar/27.1.29 9847135 PMC148090

[31] Cox J Mann M. Maxquant enables high peptide identification rates, individualized p.p.b.-range mass accuracies and proteome-wide protein quantification. Nat Biotechnol. (2008) 26:1367–72. 10.1038/nbt.1511 19029910

[32] Cox J Hein MY Luber CA Paron I Nagaraj N Mann M. Accurate proteome-wide label-free quantification by delayed normalization and maximal peptide ratio extraction, termed maxlfq. Mol Cell Proteomics (2014) 13:2513–26. 10.1074/mcp.M113.031591 24942700 PMC4159666

[33] Gravallese EM Manning C Tsay A Naito A Pan C Amento E. Synovial tissue in rheumatoid arthritis is a source of osteoclast differentiation factor. Arthritis Rheum. (2000) 43:250–8. 10.1002/1529-0131(200002)43:2<250::AID-ANR3>3.0.CO;2-P 10693863

[34] Shigeyama Y Pap T Kunzler P Simmen BR Gay RE Gay S. Expression of osteoclast differentiation factor in rheumatoid arthritis. Arthritis Rheum. (2000) 43:2523–30. 10.1002/1529-0131(200011)43:11<2523::AID-ANR20>3.0.CO;2-Z 11083276

[35] Takayanagi H Iizuka H Juji T Nakagawa T Yamamoto A Miyazaki T. Involvement of receptor activator of nuclear factor κB ligand/osteoclast differentiation factor in osteoclastogenesis from synoviocytes in rheumatoid arthritis. Arthritis Rheum. (2000) 43:259–69. 10.1002/1529-0131(200002)43:2<259::AID-ANR4>3.0.CO;2-W 10693864

[36] Cohen SB Dore RK Lane NE Ory PA Peterfy CG Sharp JT. Denosumab treatment effects on structural damage, bone mineral density, and bone turnover in rheumatoid arthritis: a twelve-month, multicenter, randomized, double-blind, placebo-controlled, phase II clinical trial. Arthritis Rheum. (2008) 58:1299–309. 10.1002/art.23417 18438830

[37] Takeuchi T Tanaka Y Ishiguro N Yamanaka H Yoneda T Ohira T. Effect of denosumab on Japanese patients with rheumatoid arthritis: a dose-response study of AMG 162 (Denosumab) in patients with rheumatoid arthritis on methotrexate to validate inhibitory effect on bone erosion (DRIVE) - A 12-month, multicentre, randomi. Ann Rheum Dis. (2016) 75:983–90. 10.1136/annrheumdis-2015-208052 26585988 PMC4893103

[38] Sharp JT Tsuji W Ory P Harper-Barek C Wang H Newmark R. Denosumab prevents metacarpal shaft cortical bone loss in patients with erosive rheumatoid arthritis. Arthritis Care Res. (2010) 62:537–44. 10.1002/acr.20172 20391509

[39] Deodhar A Dore RK Mandel D Schechtman J Shergy W Trapp R. Denosumab-mediated increase in hand bone mineral density associated with decreased progression of bone erosion in rheumatoid arthritis patients. Arthritis Care Res. (2010) 62:569–74. 10.1002/acr.20004 20391513

[40] Redlich K Hayer S Ricci R David J Tohidast-Akrad M Kollias G. Osteoclasts are essential for TNF-alpha-mediated joint destruction. J Clin Invest. (2002) 110:1419–27. 10.1172/JCI15582 12438440 PMC151809

[41] Li J Sarosi I Yan XQ Morony S Capparelli C Tan HL. RANK is the intrinsic hematopoietic cell surface receptor that controls osteoclastogenesis and regulation of bone mass and calcium metabolism. Proc Natl Acad Sci USA. (2000) 97:1566–71. 10.1073/pnas.97.4.1566 10677500 PMC26475

[42] Li P Schwarz EM O'Keefe RJ Ma L Boyce BF Xing L RANK signaling is not required for TNFalpha-mediated increase in CD11(hi) osteoclast precursors but is essential for mature osteoclast formation in TNFalpha-mediated inflammatory arthritis. J Bone Miner Res. (2004) 19:207–13. 10.1359/JBMR.0301233 14969390

[43] O'Brien W Fissel BM Maeda Y Yan J Ge X Gravallese EM. RANK-independent osteoclast formation and bone erosion in inflammatory arthritis. Arthritis Rheumatol. (2016) 68:2889–900. 10.1002/art.39837 27563728 PMC5125876

[44] Lipsky PE Davis LS Cush JJ Oppenheimer-Marks N. The role of cytokines in the pathogenesis of rheumatoid arthritis. Springer Semin Immunopathol. (1989) 11:123–62. 10.1007/BF00197186 2479111

[45] Poubelle PE Chakravarti A Fernandes MJ Doiron K Marceau AA. Differential expression of RANK, RANK-L, and osteoprotegerin by synovial fluid neutrophils from patients with rheumatoid arthritis and by healthy human blood neutrophils. Arthritis Res Ther. (2007) 9:R25. 10.1186/ar2137 17341304 PMC1906801

[46] Quercioli A Mach F Bertolotto M Lenglet S Vuilleumier N Galan K. Receptor activator of NF-κB ligand (RANKL) increases the release ofneutrophil products associated with coronary vulnerability. Thromb Haemost. (2012) 107:124–39. 10.1160/TH11-05-0324 22116393

[47] Carbone F Crowe LA Roth A Burger F Lenglet S Braunersreuther V. Treatment with anti-RANKL antibody reduces infarct size and attenuates dysfunction impacting on neutrophil-mediated injury. J Mol Cell Cardiol. (2016) 94:82–94. 10.1016/j.yjmcc.2016.03.013 27056420

[48] Park Y-J Chung MK Hwang D Kim W-U. Proteomics in rheumatoid arthritis research. Immune Netw. (2015) 15:177–85. 10.4110/in.2015.15.4.177 26330803 PMC4553255

[49] Fearon U Canavan M Biniecka M Veale DJ. Hypoxia, mitochondrial dysfunction and synovial invasiveness in rheumatoid arthritis. Nat Rev Rheumatol. (2016) 12:385–97. 10.1038/nrrheum.2016.69 27225300

[50] Potempa J Korzus E Travis J. The serpin superfamily of proteinase inhibitors: structure, function, and regulation. J Biol Chem. (1994) 269:15957–60. 10.1021/ed056pA86.2 8206889

[51] Ek-Rylander B Flores M Wendel M Heinegard D Andersson G. Dephosphorylation of osteopontin and bone sialoprotein by osteoclastic tartrate-resistant acid phosphatase. Modulation of osteoclast adhesion *in vitro*. J Biol Chem. (1994) 269:14853–6. 10.1111/j.1095-8649.2004.00473.x 8195113

[52] Bossard MJ Tomaszek TA Thompson SK Amegadzie BY Hanning CR Jones C. Proteolytic activity of human osteoclast cathepsin K: expression, purification, activation, and substrate identification. J Biol Chem. (1996) 271:12517–24. 10.1074/jbc.271.21.12517 8647860

[53] Asagiri M Hirai T Kunigami T Kamano S Gober HJ Okamoto K. Cathepsin K-dependent toll-like receptor 9 signaling revealed in experimental arthritis. Science (2008) 319:624–7. 10.1126/science.1150110 18239127

[54] Cotter K Stransky L McGuire C Forgac M. Recent insights into the structure, regulation, and function of the V-ATPases. Trends Biochem Sci. (2015) 40:611–22. 10.1016/j.tibs.2015.08.005 26410601 PMC4589219

[55] Trombetta ES Ebersold M Garrett W Pypaert M Mellman I. Activation of lysosomal function during dendritic cell maturation. Science (2003) 299:1400–3. 10.1126/science.1080106 12610307

[56] Qin A Cheng TS Pavlos NJ Lin Z Dai KR Zheng MH. V-ATPases in osteoclasts: structure, function and potential inhibitors of bone resorption. Int J Biochem Cell Biol. (2012) 44:1422–35. 10.1016/j.biocel.2012.05.014 22652318

[57] Gilman-Sachs A Tikoo A Akman-Anderson L Jaiswal M Ntrivalas E Beaman K. Expression and role of a2 vacuolar-ATPase (a2V) in trafficking of human neutrophil granules and exocytosis. J Leukoc Biol. (2015) 97:1121–31. 10.1189/jlb.3A1214-620RR 25877929

[58] Scherer O Steinmetz H Kaether C Weinigel C Barz D Kleinert H. Targeting V-ATPase in primary human monocytes by archazolid potently represses the classical secretion of cytokines due to accumulation at the endoplasmic reticulum. Biochem Pharmacol. (2014) 91:490–500. 10.1016/j.bcp.2014.07.028 25107704

[59] Ibáñez L Abou-Ezzi G Ciucci T Amiot V Belaïd N Obino D Inflammatory Osteoclasts Prime TNFα-Producing CD4+T Cells and Express CX3CR1. J Bone Miner Res. (2016) 31:1899–908. 10.1002/jbmr.2868 27161765

[60] Lotze MT Tracey KJ. High-mobility group box 1 protein (HMGB1): nuclear weapon in the immune arsenal. Nat Rev Immunol. (2005) 5:331–42. 10.1038/nri1594 15803152

[61] Yanai H Ban T Wang Z Choi MK Kawamura T Negishi H. HMGB proteins function as universal sentinels for nucleic-acid-mediated innate immune responses. Nature (2009) 462:99–103. 10.1038/nature08512 19890330

[62] Andersson U Tracey KJ. HMGB1 is a therapeutic target for sterile inflammation and infection. Annu Rev Immunol. (2011) 29:139–62. 10.1146/annurev-immunol-030409-101323 21219181 PMC4536551

[63] Taniguchi N Kawakami Y Maruyama I Lotz M. HMGB proteins and arthritis. Hum Cell (2018) 31:1–9. 10.1007/s13577-017-0182-x 28916968 PMC6541443

[64] Park JS Svetkauskaite D He Q Kim JY Strassheim D Ishizaka A. Involvement of toll-like receptors 2 and 4 in cellular activation by high mobility group box 1 protein. J Biol Chem. (2004) 279:7370–7. 10.1074/jbc.M306793200 14660645

[65] Millevoi S Loulergue C Dettwiler S Karaa SZ Keller W Antoniou M. An interaction between U2AF 65 and CF Imlinks the splicing and 3′ end processing machineries. EMBO J. (2006) 25:4854–64. 10.1038/sj.emboj.7601331 17024186 PMC1618107

[66] Whisenant TC Peralta ER Aarreberg LD Gao NJ Head SR Ordoukhanian P. The activation-induced assembly of an RNA/protein interactome centered on the splicing factor U2AF2 regulates gene expression in human CD4 T cells. PLoS ONE (2015) 10:e0144409. 10.1371/journal.pone.0144409 26641092 PMC4671683

[67] Yang W-H Ding M-J Cui G-Z Yang M Dai D-L. Heterogeneous nuclear ribonucleoprotein M promotes the progression of breast cancer by regulating the axin/β-catenin signaling pathway. Biomed Pharmacother. (2018) 105:848–55. 10.1016/j.biopha.2018.05.014 30021377

[68] Gruber JJ Zatechka DS Sabin LR Yong J Lum JJ Kong M. Ars2 Links the nuclear cap-binding complex to RNA interference and cell proliferation. Cell (2009) 138:328–39. 10.1016/j.cell.2009.04.046 19632182 PMC2717034

[69] Elahi S Egan SM Holling GA Kandefer RL Nemeth MJ Olejniczak SH. The RNA binding protein Ars2 supports hematopoiesis at multiple levels. Exp Hematol. (2018) 64:45–58.e9. 10.1016/j.exphem.2018.05.001 29775646 PMC6103522

[70] Steimer L Klostermeier D. RNA helicases in infection and disease. RNA Biol. (2012) 9:751–71. 10.4161/rna.20090 22699555

[71] Xing YH Yao RW Zhang Y Guo CJ Jiang S Xu G. SLERT regulates DDX21 rings associated with Pol I transcription. Cell (2017) 169:664–78.e16. 10.1016/j.cell.2017.04.011 28475895

[72] Zhang H-X Liu Z-X Sun Y-P Zhu J Lu S-Y Liu X-S Rig-I regulates NF- B activity through binding to Nf- b1 3′-UTR mRNA. Proc Natl Acad Sci USA. (2013) 110:6459–64. 10.1073/pnas.1304432110 23553835 PMC3631665

[73] Jiang Y Tian M Lin W Wang X Wang X. Protein kinase serine/threonine kinase 24 positively regulates interleukin 17-induced inflammation by promoting IKK complex activation. Front Immunol. (2018) 9:1–16. 10.3389/fimmu.2018.00921 29760709 PMC5936754

[74] Salzer E Santos-Valente E Keller B Warnatz K Boztug K. Protein kinase C δ: a gatekeeper of immune homeostasis. J Clin Immunol. (2016) 36:631–40. 10.1007/s10875-016-0323-0 27541826 PMC5018258

[75] Bartuzi P Hofker MH van de Sluis B. Tuning NF-κB activity: a touch of COMMD proteins. Biochim Biophys Acta (2013) 1832:2315–21. 10.1016/j.bbadis.2013.09.014 24080195

[76] Delestre-Delacour C Carmon O Laguerre F Estay-Ahumada C Courel M Elias S. Myosin 1b and F-actin are involved in the control of secretory granule biogenesis. Sci Rep. (2017) 7:5172. 10.1038/s41598-017-05617-1 28701771 PMC5507975

[77] Egami Y Fujii M Kawai K Ishikawa Y Fukuda M Araki N. Activation-inactivation cycling of Rab35 and ARF6 is required for phagocytosis of zymosan in RAW264 macrophages. J Immunol Res. (2015) 2015:429439. 10.1155/2015/429439 26229970 PMC4502309

[78] Hoffmann C Mao X Dieterle M Moreau F Absi A Al Steinmetz A. CRP2, a new invadopodia actin bundling factor critically promotes breast cancer cell invasion and metastasis. Oncotarget (2016) 7:13688–705. 10.18632/oncotarget.7327 26883198 PMC4924671

[79] Yuan H Gupte R Zelkha S Amar S. Receptor activator of nuclear factor kappa B ligand antagonists inhibit tissue inflammation and bone loss in experimental periodontitis. J Clin Periodontol. (2011) 38:1029–36. 10.1111/j.1600-051X.2011.01780.x 22092474

