# Supplementary material for: New Insights for RANKL as a Proinflammatory Modulator in Modeled Inflammatory Arthritis
Source: Front Immunol. 2019 Feb 5;10:97. doi: 10.3389/fimmu.2019.00097 (PMC6370657; doi:10.3389/fimmu.2019.00097)
Supplement: Supplementary file 1 [file Data_Sheet_1.docx]

**Supplementary Table 1: Enrichment analysis of Cluster I based on KEGG pathways.** For each pathway the Benjamini-corrected p value, % pathway association, the number of genes (Nr. Genes), and the associated genes found are provided.

| **GOTerm** | **p-value** | **Associated Genes (%)** | **Nr. Genes** | **Associated Genes** |
| --- | --- | --- | --- | --- |
| Citrate cycle (TCA cycle) | 2.54E-20 | 53.13 | 17 | Aco2, Cs, Fh1, Idh2, Idh3a, Idh3b, Idh3g, Mdh1, Mdh2, Ogdh, Pcx, Pdha1, Pdhb, Sdha, Sucla2, Suclg1, Suclg2 |
| Parkinson disease | 2.61E-16 | 17.36 | 25 | Atp5a1, Atp5b, Atp5o, Cox7a1, Cycs, Ndufa2, Ndufb4, Ndufc2, Ndufs1, Ndufs2, Ndufs4, Ndufs7, Ndufv1, Park7, Prkaca, Sdha, Slc25a4, Snca, Uchl1, Uqcrb, Uqcrc1, Uqcrc2, Uqcrfs1, Vdac1, Vdac3 |
| Fatty acid degradation | 4.76E-15 | 32.00 | 16 | Acaa2, Acadl, Acadm, Acadvl, Acat1, Acsl1, Acsl6, Adh1, Aldh2, Cpt1b, Echs1, Eci1, Eci2, Hadh, Hadha, Hadhb |
| Pyruvate metabolism | 3.21E-14 | 36.84 | 14 | Acat1, Acyp2, Aldh2, Fh1, Grhpr, Ldha, Ldhb, Mdh1, Mdh2, Me1, Pcx, Pdha1, Pdhb, Pkm |
| Thermogenesis | 3.26E-14 | 12.17 | 28 | Acsl1, Acsl6, Atp5a1, Atp5b, Atp5j2, Atp5k, Atp5o, Cox7a1, Cpt1b, Gnas, Lipe, Mapk12, Mgll, Ndufa2, Ndufb4, Ndufc2, Ndufs1, Ndufs2, Ndufs4, Ndufs7, Ndufv1, Plin1, Prkaca, Sdha, Uqcrb, Uqcrc1, Uqcrc2, Uqcrfs1 |
| Alzheimer disease | 2.01E-13 | 13.71 | 24 | Atp2a1, Atp2a2, Atp2a3, Atp5a1, Atp5b, Atp5o, Cacna1s, Cox7a1, Cycs, Ndufa2, Ndufb4, Ndufc2, Ndufs1, Ndufs2, Ndufs4, Ndufs7, Ndufv1, Ppp3ca, Sdha, Snca, Uqcrb, Uqcrc1, Uqcrc2, Uqcrfs1 |
| Oxidative phosphorylation | 5.12E-12 | 14.93 | 20 | Atp5a1, Atp5b, Atp5j2, Atp5k, Atp5o, Cox7a1, Ndufa2, Ndufb4, Ndufc2, Ndufs1, Ndufs2, Ndufs4, Ndufs7, Ndufv1, Ppa1, Sdha, Uqcrb, Uqcrc1, Uqcrc2, Uqcrfs1 |
| Glycolysis / Gluconeogenesis | 1.24E-10 | 20.90 | 14 | Adh1, Aldh2, Aldoa, Hk2, Ldha, Ldhb, Pdha1, Pdhb, Pfkm, Pgam2, Pgk1, Pgm2, Pkm, Tpi1 |

**Supplementary Table 2: Enrichment analysis of Cluster II based on KEGG pathways.** For each pathway the Benjamini-corrected p value, % pathway association, the number of genes (Nr. Genes), and the associated genes found are provided.

| **GOTerm** | **p-value** | **Associated Genes (%)** | **Nr. Genes** | **Associated Genes** |
| --- | --- | --- | --- | --- |
| Ribosome | 6.75E-44 | 22.29 | 39 | Rpl10a, Rpl13, Rpl13a, Rpl14, Rpl17, Rpl18, Rpl18a, Rpl22, Rpl23, Rpl23a, Rpl24, Rpl26, Rpl27a, Rpl3, Rpl30, Rpl32, Rpl36, Rpl37a, Rpl6, Rpl7, Rpl8, Rps10, Rps11, Rps15a, Rps16, Rps18, Rps19, Rps2, Rps24, Rps25, Rps26, Rps3, Rps3a1, Rps4x, Rps5, Rps6, Rps8, Rps9, Rpsa |
| Protein processing in endoplasmic reticulum | 2.83E-12 | 9.82 | 16 | Ckap4, Cul1, Dad1, Ddost, Dnajb11, Hspa5, Rpn1, Rpn2, Rrbp1, Sec24d, Sec31a, Sec61a1, Ssr1, Ssr4, Txndc5, Ube2d3 |
| Protein export | 2.58E-07 | 21.43 | 6 | Hspa5, Sec61a1, Spcs3, Srp54a, Srp68, Srp72 |
| Aminoacyl-tRNA biosynthesis | 3.22E-05 | 9.09 | 6 | Dars, Eprs, Gars, Iars, Lars, Nars |
| Arginine and proline metabolism | 9.97E-05 | 10.00 | 5 | Aldh18a1, Ckb, P4ha1, P4ha2, Pycr1 |

**Supplementary Table 3: Enrichment analysis of Cluster III based on KEGG pathways.** For each pathway the Benjamini-corrected p value, % pathway association, the number of genes (Nr. Genes), and the associated genes found are provided.

| **GOTerm** | **p-value** | **Associated Genes (%)** | **Nr. Genes** | **Associated Genes** |
| --- | --- | --- | --- | --- |
| Phagosome | 1.02E-19 | 18.23 | 33 | Actg1, Atp6v0d1, Atp6v1a, Atp6v1b2, Atp6v1d, Atp6v1e1, Atp6v1g1, Atp6v1h, C3, Canx, Coro1a, Ctss, Cybb, Dync1h1, H2-D1, Itga5, Itgam, Itgav, Itgb2, Lamp1, Mbl2, Mpo, Mrc1, Ncf1, Ncf2, Ncf4, Rab5c, Rab7, Sec61b, Stx7, Tcirg1, Tubb5, Tubb6 |
| Lysosome | 2.63E-14 | 18.55 | 23 | Acp5, Ap1m1, Ap3d1, Asah1, Atp6v0d1, Atp6v1h, Cltc, Ctsa, Ctsb, Ctsc, Ctsd, Ctsh, Ctss, Ctsz, Glb1, Hexa, Hexb, Lamp1, Lgmn, Man2b1, Naga, Ppt1, Tcirg1 |
| Proteasome | 3.07E-13 | 32.61 | 15 | Psma1, Psma2, Psma3, Psma4, Psma5, Psma6, Psma7, Psmb1, Psmb2, Psmb4, Psmb7, Psmb8, Psmc2, Psme1, Psme2 |
| Regulation of actin cytoskeleton | 4.47E-11 | 11.63 | 25 | Actg1, Actn1, Arhgef1, Arpc1b, Arpc2, Arpc3, Arpc4, Arpc5, Cfl1, Cyfip1, Ezr, F2, Fn1, Iqgap1, Iqgap2, Itga5, Itgam, Itgav, Itgb2, Mapk3, Msn, Myh9, Myl12a, Nckap1, Nckap1l |
| Leukocyte transendothelial migration | 3.01E-10 | 15.65 | 18 | Actg1, Actn1, Cybb, Ezr, Gnai2, Itgam, Itgb2, Mapk14, Mmp9, Msn, Myl12a, Ncf1, Ncf2, Ncf4, Plcg2, Rap1b, Thy1, Vasp |
| Bacterial invasion of epithelial cells | 3.06E-10 | 20.27 | 15 | Actg1, Arpc1b, Arpc2, Arpc3, Arpc4, Arpc5, Cltc, Dnm2, Elmo1, Fn1, Hcls1, Itga5, Rhog, Sept8, Sept9 |
| Fc gamma R-mediated phagocytosis | 2.43E-09 | 17.24 | 15 | Arpc1b, Arpc2, Arpc3, Arpc4, Arpc5, Cfl1, Dnm2, Dock2, Mapk3, Marcks, Ncf1, Plcg2, Prkcd, Ptprc, Vasp |
| Salmonella infection | 5.10E-09 | 17.95 | 14 | Actg1, Arpc1b, Arpc2, Arpc3, Arpc4, Arpc5, Dync1h1, Flna, Lbp, Mapk14, Mapk3, Pycard, Rab7, Rhog |

**Supplementary Table 4: Enrichment analysis of proteins downregulated in the ankles from Tg197/Tg5519 mice compared to those isolated from Tg197 mice based on KEGG pathways.** For each pathway the Benjamini-corrected p value, % pathway association, the number of genes (Nr. Genes), and the associated genes found are provided.

| **GOTerm** | **p-value** | **Associated Genes (%)** | **Nr. Genes** | **Associated Genes** |
| --- | --- | --- | --- | --- |
| Starch and sucrose metabolism | 1.77E-04 | 18.18 | 6 | Gbe1, Gpi1, Pgm1, Pgm2l1, Pygb, Pygm |
| Glycolysis / Gluconeogenesis | 1.99E-04 | 11.94 | 8 | Gpi1, Pfkm, Pgam2, Pgk1, Pgm1, Pkm, Tpi1 |
| Complement and proteinase inhibitors | 1.09E-03 | 9.09 | 8 | A2m, Cfi, Kng1, Mbl2, Serpina1b, Serpina1c, Serpina1d, Serpinc1 |
| PPAR signaling pathway | 1.20E-02 | 7.06 | 6 | Acadl, Cpt1b, Fabp3, Fabp4, Me1 |
| Muscle contraction | 2.60E-02 | 6.41 | 5 | Cacna2d1, Cacng1, Cox7a1, Tpm3 |

**Supplementary Table 5: Enrichment analysis of proteins upregulated in the ankles from Tg197/Tg5519 mice compared to those isolated from Tg197 mice based on KEGG pathways.** For each pathway the Benjamini-corrected p value, % pathway association, the number of genes (Nr. Genes), and the associated genes found are provided.

| **GOTerm** | **p-value** | **Associated Genes (%)** | **Nr. Genes** | **Associated Genes** |
| --- | --- | --- | --- | --- |
| Rheumatoid arthritis | 1.11E-05 | 13.25 | 11 | Acp5, Atp6v0d1, Atp6v0d2, Atp6v1a, Atp6v1b2, Atp6v1d, Atp6v1e1, Atp6v1g1, Atp6v1h, Ctsk, Tcirg1 |
| Protein processing in endoplasmic reticulum | 2.89E-05 | 8.59 | 14 | Cul1, Dad1, Erp29, Plaa, Preb, Sec24d, Sec31a, Sec61a1, Stt3a, Ube2d3 |
| Glycine, serine and threonine metabolism | 4.24E-04 | 15.00 | 6 | Gcsh, Maoa, Psat1, Shmt2 |
| Arginine and proline metabolism | 6.44E-03 | 10.00 | 5 | Aldh18a1, Ckb, Maoa, Sms |
| Ribosome | 1.41E-02 | 5.14 | 9 | Rpl13a, Rpl23, Rpl27a, Rpl30, Rpl32, Rpl35, Rps19, Rps6, Rps9 |

**Supplementary Table 6: Proteins with significant reduced expression in the ankles from Tg197/Tg5519 mice compared to those isolated from their littermates Tg197, and Tg5519.** Logarithmic LFQ mean values are provided for each genotype.

| **Gene Name** | **Protein ID** | **Topology** | **Anova**  **p-value** | **WT** | **Tg5519** | **Tg197** | **Tg197/**  **Tg5519** |
| --- | --- | --- | --- | --- | --- | --- | --- |
| **Lipid metabolism** | | | | | | | |
| Acadl | P51174 | Mitochondrion | 2.3E-04 | 23.15 | 22.90 | 23.04 | 22.49 |
| Ces1d | Q8VCT4 | Endoplasmic reticulum  Cytoplasm | 2.9E-05 | 20.86 | 19.97 | 19.82 | 19.06 |
| Cpt1b | Q924X2 | Mitochondrion | 1.0E-07 | 20.79 | 20.66 | 20.06 | 19.48 |
| Eci1 | P42125 | Mitochondrion | 1.9E-06 | 21.94 | 21.79 | 21.79 | 21.32 |
| Fabp3 | P11404 | Cytoplasm | 5.1E-07 | 22.30 | 21.48 | 21.04 | 20.18 |
| Fabp4 | P04117 | Nucleus  Cytoplasm | 2.4E-04 | 25.93 | 25.54 | 25.64 | 24.86 |
| Me1 | P06801 | Cytoplasm | 2.0E-03 | 21.04 | 20.91 | 21.00 | 20.36 |
| **Aminoacid / Nucleotide metabolism** | | | | | | | |
| Adsl | P54822 | Cytosol  Mitochondrion | 5.5E-06 | 21.95 | 21.69 | 21.53 | 21.24 |
| Ak1 | Q9R0Y5 | Cytoplasm | 1.5E-09 | 26.05 | 25.89 | 25.44 | 24.94 |
| Ckmt2 | Q6P8J7 | Mitochondrion | 1.6E-07 | 23.29 | 23.42 | 22.71 | 21.96 |
| Ampd1 | Q3V1D3 | Cytosol | 2.6E-07 | 23.05 | 23.09 | 22.56 | 21.84 |
| Hibadh | Q99L13 | Mitochondrion | 3.1E-05 | 21.16 | 21.09 | 20.87 | 20.59 |
| Mpst | Q99J99 | Mitochondrion | 1.6E-07 | 20.21 | 20.27 | 20.07 | 19.74 |
| Nqo2 | Q9JI75 | Cytoplasm | 5.1E-03 | 19.10 | 0.00 | 18.98 | 18.13 |
| Nt5c3a | Q9D020 | Cytoplasm | 6.6E-10 | 21.66 | 21.11 | 20.67 | 20.23 |
| Qdpr | Q8BVI4 | Cytosol Mitochondrion | 8.2E-04 | 21.28 | 21.14 | 21.02 | 20.58 |
| **Carbohydrate metabolism** | | | | | | | |
| Gbe1 | Q9D6Y9 | Cytosol | 4.0E-07 | 21.66 | 21.56 | 21.01 | 20.44 |
| Gpd1 | P13707 | Cytoplasm | 3.1E-08 | 23.68 | 23.96 | 23.23 | 22.72 |
| Gpd2 | Q64521 | Mitochondrion | 1.7E-03 | 21.48 | 21.52 | 21.38 | 21.00 |
| Pfkm | P47857 | Cytoplasm | 4.2E-07 | 25.91 | 26.08 | 25.31 | 24.69 |
| Pgk1 | P09411 | Cytoplasm | 6.6E-09 | 26.65 | 26.66 | 26.22 | 26.04 |
| Pgm1 | Q9D0F9 | Cytoplasm | 2.4E-11 | 25.63 | 25.11 | 25.00 | 24.31 |
| Pgp | Q8CHP8 | Cytoplasm | 4.0E-05 | 20.26 | 20.50 | 20.06 | 19.71 |
| Phpt1 | Q9DAK9 | Nucleus  Cytoplasm | 3.5E-04 | 20.51 | 20.58 | 20.37 | 19.96 |
| Pkm | P52480 | Cytosol | 7.4E-06 | 28.12 | 28.32 | 27.72 | 27.47 |
| Pygm | Q9WUB3 | Endoplasmic reticulum | 1.6E-05 | 25.82 | 26.29 | 25.45 | 24.85 |
| Tpi1 | P17751 | Cytosol | 4.5E-10 | 27.14 | 27.00 | 26.64 | 26.10 |
| **Mitochondria - Energy production** | | | | | | | |
| Atp5j | P97450 | Mitochondrion | 2.2E-03 | 21.93 | 0.00 | 21.67 | 20.89 |
| Cox7a1 | P56392 | Mitochondrion | 9.9E-06 | 20.61 | 20.67 | 20.28 | 19.90 |
| D10Jhu81e | Q9D172 | Mitochondrion | 2.6E-07 | 22.10 | 21.66 | 21.57 | 21.07 |
| Fh | P97807 | Mitochondrion | 1.2E-06 | 23.79 | 23.76 | 23.53 | 23.01 |
| Mpc2 | Q9D023 | Mitochondrion | 4.2E-04 | 19.72 | 20.19 | 19.85 | 19.09 |
| **Muscle Function** | | | | | | | |
| Atp2a3 | Q64518 | Endoplasmic and Sarcoplasmic reticulum | 2.2E-04 | 25.59 | 25.35 | 25.00 | 23.49 |
| Bin1 | O08539 | Nucleus  Cytoplasm | 4.3E-08 | 23.53 | 23.52 | 22.88 | 22.31 |
| Cacng1 | O70578 | Sarcolemma | 3.8E-02 | 18.23 | 18.48 | 18.50 | 17.65 |
| Jsrp1 | Q3MI48 | Endoplasmic reticulum | 2.7E-05 | 20.18 | 19.78 | 19.46 | 18.83 |
| Myh4 | Q5SX39 | Myofibril | 7.7E-04 | 20.00 | 22.10 | 20.81 | 18.57 |
| Myh8 | B2RWW8 | Myosin complex | 6.0E-04 | 20.34 | 20.98 | 20.81 | 19.75 |
| Myom1 | Q62234 | Cytoskeleton | 1.8E-05 | 23.39 | 23.50 | 23.09 | 22.31 |
| Smtnl2 | Q8CI12 | Cytoskeleton | 3.4E-07 | 20.08 | 19.47 | 19.05 | 18.41 |
| Srl | Q7TQ48 | Sarcoplasmic reticulum | 1.7E-08 | 26.32 | 26.44 | 25.80 | 25.22 |
| Tnnc2 | P20801 | Cytoskeleton | 5.3E-03 | 22.73 | 0.00 | 22.67 | 21.25 |
| Trim72 | Q1XH17 | Sarcolemma | 6.7E-09 | 22.82 | 22.85 | 22.01 | 21.36 |
| **Cytoskeleton** | | | | | | | |
| Cfl2 | P45591 | Cytoskeleton | 1.0E-05 | 23.98 | 23.96 | 23.37 | 22.30 |
| Mybpc1 | D3YU50 | Cytoskeleton | 3.8E-02 | 20.06 | 0.00 | 19.74 | 18.40 |
| Mybph | P70402 | Cytoskeleton | 2.6E-09 | 21.15 | 21.40 | 19.80 | 18.80 |
| Sptbn1 | Q62261 | Cytoskeleton | 8.1E-03 | 24.53 | 24.55 | 24.46 | 24.13 |
| Tuba4a | A0A087WSL5 | Cytoskeleton | 5.9E-07 | 22.50 | 22.60 | 21.84 | 21.37 |
| **DNA, RNA and Protein processing** | | | | | | | |
| Cryab | P23927 | Nucleus  Cytoplasm | 4.0E-08 | 24.97 | 25.06 | 23.94 | 23.48 |
| Eef1a2 | P62631 | Nucleus | 8.6E-05 | 24.40 | 24.92 | 23.85 | 23.20 |
| Eif4a2 | P10630 | Nucleus | 1.3E-03 | 19.17 | 19.17 | 18.86 | 18.12 |
| Hspb2 | Q99PR8 | Nucleus  Cytoplasm | 6.1E-07 | 21.12 | 20.05 | 20.16 | 19.26 |
| Lmcd1 | Q8VEE1 | Nucleus | 8.3E-14 | 23.00 | 22.57 | 21.50 | 21.05 |
| Naca | Q60817 | Nucleus  Cytoplasm | 4.6E-11 | 23.43 | 23.50 | 22.35 | 21.85 |
| Ube2k | P61087 | Cytoplasm | 2.7E-06 | 19.19 | 19.06 | 19.30 | 18.25 |
| Zmpste24 | Q80W54 | Endoplasmic reticulum | 4.6E-04 | 18.87 | 18.26 | 19.19 | 18.32 |
| **Protease inhibitors** | | | | | | | |
| Kng1 | O08677 | Extracellular region Secreted | 2.1E-04 | 21.53 | 20.45 | 21.80 | 20.62 |
| Serpina1b | P22599 | Extracellular region Secreted | 3.4E-03 | 23.12 | 23.03 | 22.93 | 21.87 |
| Serpina1c | Q00896 | Extracellular region /Secreted | 3.0E-03 | 24.79 | 24.93 | 24.45 | 23.47 |
| Serpina1d | Q00897 | Extracellular region Secreted | 6.0E-05 | 26.10 | 25.97 | 25.75 | 25.14 |
| Serpinc1 | P32261 | Extracellular region Secreted | 4.8E-04 | 21.68 | 21.65 | 21.60 | 21.17 |
| **Miscellaneous** | | | | | | | |
| Cav1 | P49817 | Golgi apparatus membrane | 6.3E-03 | 21.47 | 21.59 | 21.65 | 19.90 |
| Ehd2 | Q8BH64 | Cytosol | 2.4E-08 | 24.66 | 24.68 | 24.25 | 23.80 |
| Mfge8 | P21956 | Extracellular region Secreted | 2.1E-06 | 21.41 | 20.70 | 20.50 | 19.84 |
| Ndrg2 | Q9QYG0 | Cytoplasm | 5.6E-07 | 24.64 | 24.57 | 23.87 | 22.87 |
